# Adaptation to the High-Arctic island environment despite long-term reduced genetic variation in Svalbard reindeer

**DOI:** 10.1016/j.isci.2023.107811

**Published:** 2023-09-03

**Authors:** Nicolas Dussex, Ole K. Tørresen, Tom van der Valk, Mathilde Le Moullec, Vebjørn Veiberg, Ave Tooming-Klunderud, Morten Skage, Benedicte Garmann-Aarhus, Jonathan Wood, Jacob A. Rasmussen, Åshild Ø. Pedersen, Sarah L.F. Martin, Knut H. Røed, Kjetill S. Jakobsen, Love Dalén, Brage B. Hansen, Michael D. Martin

**Affiliations:** 1 Department of Natural History, University Museum, Norwegian University of Science and Technology (NTNU), Erling Skakkes gate 47A, Trondheim, Norway; 2 Centre for Ecological and Evolutionary Synthesis (CEES), Department of Biosciences, University of Oslo, PO Box 1066 Blindern, N-0316 Oslo, Norway; 3 Centre for PalaeoGenetics, Svante Arrhenius väg 20C, SE 106 91 Stockholm, Sweden; 4 Department of Bioinformatics and Genetics, Swedish Museum of Natural History, SE 104 05 Stockholm, Sweden; 5 Centre for Biodiversity Dynamics, Department of Biology, Norwegian University of Science and Technology (NTNU), NO 7491 Trondheim, Norway; 6 Department of Terrestrial Ecology, Norwegian Institute for Nature Research (NINA), NO 7034 Trondheim, Trondheim, Norway; 7 Natural History Museum, University of Oslo, NO 0318 Oslo, Norway; 8 Tree of Life, Wellcome Sanger Institute, Wellcome Genome Campus, Hinxton CB10 1SA Cambridge, UK; 9 Globe Institute, University of Copenhagen, 2100 Copenhagen, Denmark; 10 Norwegian Polar Institute, Fram Centre, NO 9296 Tromsø, Norway; 11 Department of Preclinical Sciences and Pathology, Norwegian University of Life Sciences, P.O. Box 5003, 1432 Ås, Norway; 12 Department of Zoology, Stockholm University, SE-106 91 Stockholm, Sweden

**Keywords:** Sequence analysis, Genomics, Animal species

## Abstract

Typically much smaller in number than their mainland counterparts, island populations are ideal systems to investigate genetic threats to small populations. The Svalbard reindeer (*Rangifer tarandus platyrhynchus*) is an endemic subspecies that colonized the Svalbard archipelago ca. 6,000–8,000 years ago and now shows numerous physiological and morphological adaptations to its arctic habitat. Here, we report a *de*-*novo* chromosome-level assembly for Svalbard reindeer and analyze 133 reindeer genomes spanning Svalbard and most of the species’ Holarctic range, to examine the genomic consequences of long-term isolation and small population size in this insular subspecies. Empirical data, demographic reconstructions, and forward simulations show that long-term isolation and high inbreeding levels may have facilitated the reduction of highly deleterious—and to a lesser extent, moderately deleterious—variation. Our study indicates that long-term reduced genetic diversity did not preclude local adaptation to the High Arctic, suggesting that even severely bottlenecked populations can retain evolutionary potential.

## Highlights

• Analyses of 133 reindeer (*Rangifer tarandus*) genomes from the Holarctic and Svalbard
• Svalbard reindeer diverged from Novaya Zemlya (Russia) ca. 6,200 years ago
• Svalbard reindeer have the highest levels of inbreeding among all reindeer subspecies
• Simulations support long-term inbreeding and load purging in Svalbard reindeer

## Graphical abstract

**Figure:**
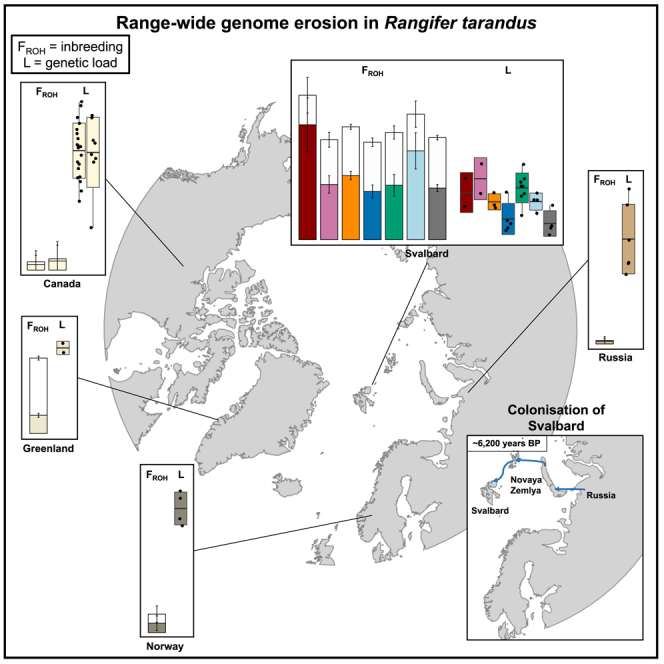

## Introduction

In the present era of unprecedented extinction rates,^1^ understanding the dynamics of genetic variation in small populations is essential to assess the extinction risk of species.^2^^,^^3^ Genome erosion is an important threat to small populations as it puts them at risk of extinction through the loss of genome-wide diversity and evolutionary potential, increases in genetic load (i.e., mutational meltdown), maladaptation (i.e., mismatch between adaptations and environmental conditions), and genetic introgression after hybridization.^4^ Understanding how small populations respond to severe population declines is thus essential for species management and recovery.

Due to their geographical isolation as well as smaller census and effective (N_e_) population sizes compared to species from larger landmasses, island-inhabiting taxa provide unique opportunities to study the effects of population bottlenecks and can thus be used as models for declining and endangered species.^5^ Indeed, island and endangered populations both have experienced severe bottlenecks throughout their evolutionary history, either through founder effects and gradual geographical isolation or through habitat degradation or human-induced declines. Many island and isolated mainland populations were founded by, or descended from, a relatively small number of individuals (n = 2–20; e.g., Isle Royale moose^6^ and wolf,^7^ Tiburon Island bighorn sheep,^8^ Swedish wolf^9^). The strong drift effect associated with severe bottlenecks will favor a reduction in genetic diversity and evolutionary potential and an increase in genetic load and frequency of harmful mutations (e.g.,^5^^,^^10^), which can increase the risk of extinction.^2^^,^^4^^,^^11^ However, consistent with theory and simulations,^12^^,^^13^ there is mounting empirical evidence that long-term isolation and small population size favor the reduction of genetic load (i.e., purging^12^^,^^13^), in particular of highly deleterious variation, via the homozygous expression of deleterious alleles and subsequent purifying selection (e.g., kākāpō,^14^ ibex,^15^ Indian tiger,^16^ Orkney voles,^17^ Channel Island foxes,^18^ Chatham Island black robin^19^).

Yet, while purging of highly deleterious load can occur, moderately or weakly deleterious variation can also increase in frequency and still threaten the long-term survival of species. Thus, the dynamics of load are complex.^11^^,^^13^ Furthermore, life-history traits (e.g., mating system, litter size) and the speed of demographic recovery will affect how fast the population can purge its genetic load and reach a new equilibrium.^20^^,^^21^ For instance, a rapid population rebound could mitigate the effects of a bottleneck and genetic drift, whereas a slow recovery would instead lead to a higher exposure of deleterious variant in homozygous state and increase the risk of inbreeding depression.^11^^,^^13^

One biological system well suited to study the evolution of load in small populations is the Svalbard reindeer (*Rangifer tarandus platyrhynchus*), a divergent, endemic reindeer subspecies that has evolved a suite of morphological and physiological adaptations (e.g., small head and body size, short extremities, ability to digest bryophytes, seasonal regulation in circadian rhythms) to the cold, insular, and seasonally extreme environment of the Svalbard archipelago in the High Arctic (79.0° N, 17.7° E).^22^^,^^23^ Reindeer feces were found in Svalbard peat deposits dated to 3,800–5,000 years before present (BP),^24^ and ancient antlers were recently dated up to 7,080 years BP (M. Le Moullec, B. B. Hansen & M. Martin, *unpubl. data*). Furthermore, ancient mitogenome data suggest that the archipelago was colonized from Russia via Novaya Zemlya and the Franz Josef Land archipelago only ca. 8,000 years BP.^25^ These dates are consistent with records of sparse vegetation from 10,000 years BP^26^ indicating that colonization before this time is unlikely. Consequently, the reindeers’ adaptation to this new environment must have occurred relatively quickly (i.e., at least within ca. 1,000 generations, assuming a generation time of six years^27^). Importantly, based on their reduced variation compared to that of mainland reindeer, the Svalbard population was probably founded by a relatively small number of individuals, followed by a gradual bottleneck as this new population became increasingly isolated from the Eurasian mainland.^28^

The subspecies faced a recent near-extinction in the 20^th^ century.^29^ Reindeer hunting in Svalbard started in the seventeenth century and ended when the practice was banned in 1925.^29^ By then, the Svalbard reindeer had been nearly extirpated from most of its original range through overharvesting.^29^ However, thanks to legislation and two reintroduction events to the western part of the archipelago (Brøggerhalvøya, Daudmannsøyra), the subspecies has now recovered and recolonized its former range and numbers ca. 20,000 individuals.^29^ Svalbard reindeer have the lowest heterozygosity yet documented in *Rangifer tarandus*,^30^ and a recent genomic study has reported differential levels of heterozygosity and inbreeding across the archipelago.^27^ There is also evidence for significant genetic drift resulting from recent reintroductions to two regions of the archipelago, but no significant decrease in heterozygosity or increase in inbreeding compared to the source population.^27^ However, there is to date no evidence for the effect of long-term insularity and of this recent overharvesting decline on genetic load. This species thus provides a unique opportunity to contrast the effects of long-term isolation and of the recent bottleneck on genetic load, and to understand how it can affect the long-term persistence of Svalbard reindeer populations.

Here, we report a *de-novo*, chromosome-level genome assembly for the Svalbard reindeer. We utilize it in a comparative genomics approach, analyzing 133 genome sequences from the Svalbard population as well as larger populations from most of the Holarctic; we examine the genomic consequences of long-term population isolation and a recent bottleneck. Our data show a reduction in highly deleterious variation on Svalbard relative to mainland subspecies with larger populations. Finally, we perform forward-in-time simulations and show that a strong founder effect associated with the colonization of Svalbard may have induced a severe increase in inbreeding and a reduction in highly deleterious genetic load. Our results indicate that in spite of a severe and long-term reduction in genetic diversity, Svalbard reindeer successfully adapted to the High-Arctic environment.

## Results

### *De-novo* assembly of reference genome

A total of 32-fold coverage in Pacific Biosciences single-molecule HiFi long reads and 56-fold coverage in Arima Hi-C reads were generated and assembled, resulting in two haplotype-separated assemblies: haplotype 1 (2,970 Mbp; N_50_ = 66 Mbp) and haplotype 2 (2,830 Mbp; N_50_ = 65 Mbp). Haplotype 1 comprised 34 autosomes as well as the X and Y chromosomes. BUSCO analyses showed 96.3% and 94.1% complete genes using the mammalian lineage set and a contig N_50_ of 22.5 Mbp (haplotype 1) and 25.5 Mbp (haplotype 2). Further details on assembly quality are given in the supplemental information.

### Past demography

**Figure 1:**
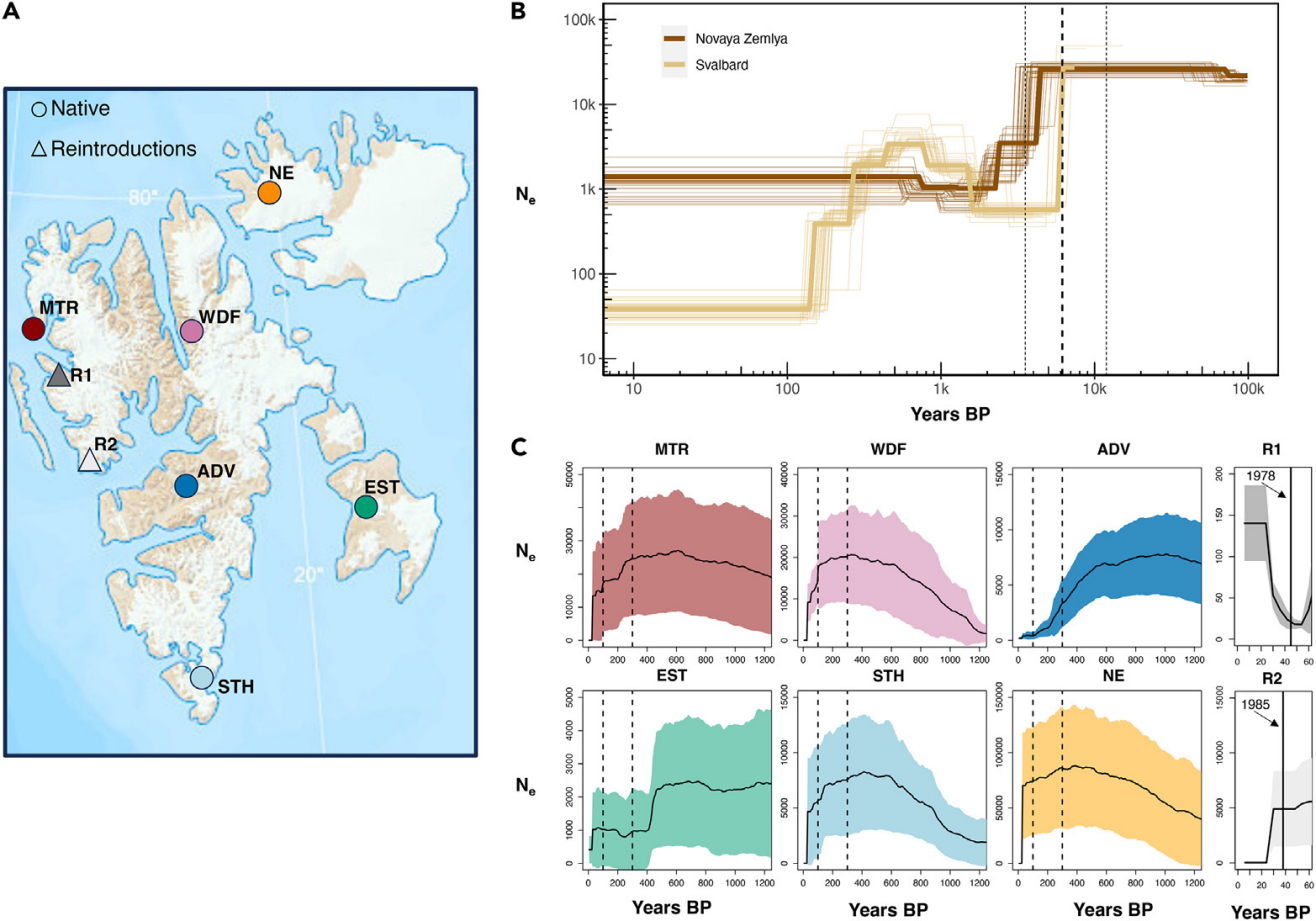
Sampling and past demography of Svalbard reindeer (A) Sampling locations of Svalbard reindeer genomes used in this study. Subpopulation coloring is based on Burnett et al.^27^ clustering. (B) Joint demography and divergence estimates between Svalbard and Russia (Novaya Zemlya archipelago) reindeer using the SMC++ and a mutation rate of 1.06 × 10e^−8^ per generation.^33^ The thick vertical dashed line represents the mean and the thin vertical dashed lines represent the 5^th^ and 95^th^ percentiles for divergence time. (C) Demographic reconstruction over the past 200 generations using the linkage disequilibrium approach implemented in GONE. Black line depicts the mean and shaded areas the 95% CI for 30 independent runs. Dashed vertical lines represent the approximate start and end of the bottleneck and full black lines, the time of reintroductions for R1 and R2. For both SMC++ and GONE, we assumed a generation time of six years.^27^

We analyzed a total of 133 reindeer individuals from most of the species’ Holarctic range, including 91 individuals from Svalbard (Figure 1A). Clustering from the principal component analysis was largely consistent with the geographical origin of samples and showed that Russian reindeer cluster most closely with Svalbard reindeer. Within Canada, we found a distinction between caribou from the Euro-Beringian and North American lineages,^31^ whereas the structure within Svalbard supported clustering into six previously described subpopulations^27^^,^^32^ (Figure S1).

Past demography reconstruction using the coalescent approach of SMC++ revealed an overall decline ca. 10,000 years BP coinciding with the divergence of Svalbard and Novaya Zemlya reindeer, which was estimated at ca. 6,200 (5^th^–95^th^ percentiles: 3,530–11,919) years BP (Figure 1B). This was followed by a period of constant N_e_ ranging between ∼500 and 600 (Figures 1B and S2). The reconstruction also showed a population increase between 1,000 and 2,000 years BP and a recent bottleneck ca. 400 years BP.

Furthermore, GONE, which is able to detect recent changes in population size, showed increases in N_e_ for most of the six subpopulations ca. 1,200 years BP (Figure 1C). There was also evidence for a severe decline dating back to ∼100–300 years BP in most subpopulations (Figure 1C). Historical N_e_ estimates varied among subpopulations with EST showing the lowest N_e_ of ∼2,000 prior to the recent decline. Among the two reintroduced subpopulations, R1 experienced a rapid increase post re-introduction, whereas R2 showed a decline.

### Inbreeding and heterozygosity

**Figure 2:**
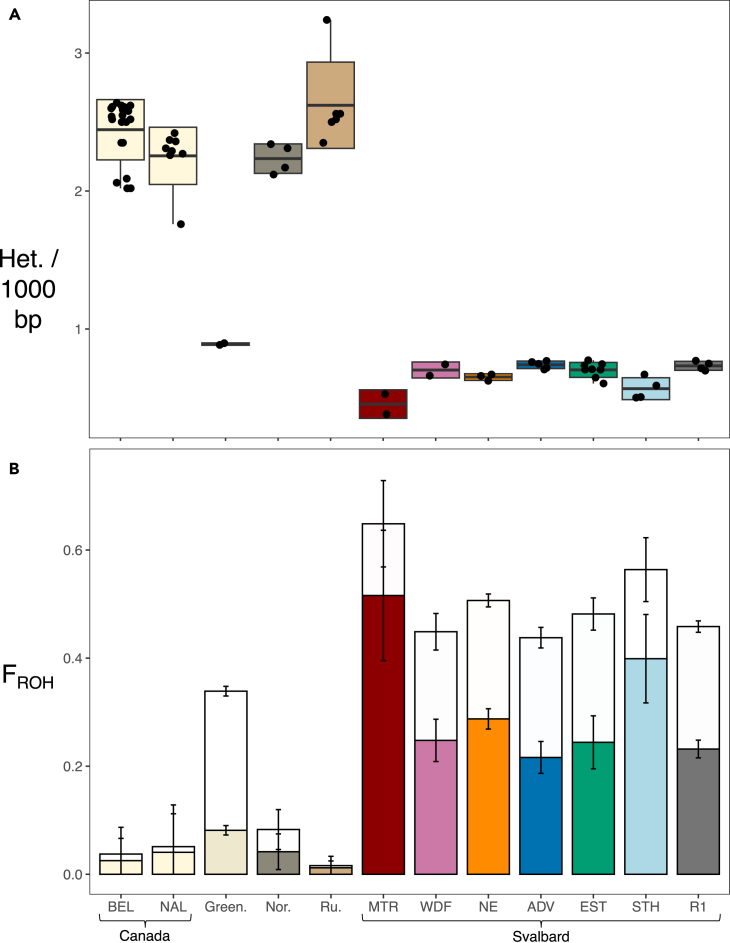
Geographical comparison of heterozygosity and inbreeding for five *Rangifer tarandus* populations (A) Heterozygosity (het. sites/1000 bp). Horizontal lines within boxplots depict the mean, bounds of boxes represent the standard deviation, and vertical bars represent minima and maxima. (B) Inbreeding coefficients (F_ROH_). Bars extending from the mean values represent the standard deviation. Complete bars show the proportion of genomes in ROH ≥ 100 kb (i.e., background relatedness) and lower coloured portions of bars show proportions in ROH ≥ 2 Mb (i.e., recent inbreeding events). Results are shown for the following parameters: *homozyg-window-snp* 1000, *homozyg-window-het* 1 (see STAR methods). (Green. = Greenland; Nor. = Norway; Ru. = Russia).

Estimates of heterozygosity and inbreeding based on the identification of runs of homozygosity (F_ROH_) showed that the Svalbard reindeer population has the lowest heterozygosity (p = 1 × 10^−6^) as well as highest inbreeding (p = 1 × 10^−6^), followed by the Western Greenland population, compared to larger populations of Canadian caribou and Russian reindeer (Figures 2 and S2). Within Svalbard, Mitrahalvøya (MTR) and Southern Spitsbergen (STH) showed the lowest mean heterozygosity and highest inbreeding.

F_ROH_ varied substantially among populations and ranged between 0.016 and 0.49 for ROH ≥ 100 kb and between 0.011 and 0.28 for ROH ≥ 2 Mb (Figure 2B and Table S1). On Svalbard, ROH ≥ 2 Mb accounted for 56% of the total F_ROH_ on average (Figure 2B and Table S1). The second most inbred population was that of Western Greenland, where the proportion of ROH ≥ 2 Mb only accounted for 24% of the total F_ROH_ on average. Maximum ROH length ranged between 15 and 74 Mb, with Svalbard genomes having the longest ROH (Figure S2).

The distribution of ROH length indicated that a large proportion of inbreeding events occurred as recently as 144 years BP in Canada, Norway, Russia, and Svalbard (i.e., ROH ≥ 2 Mb; Figures 2, S2, and Table 1). In Svalbard, the presence of ROH ranging from 30 to 80 Mb supported even more recent inbreeding up to four years BP (Figure S2 and Table 1). In contrast, Western Greenland genomes showed a larger proportion of shorter ROH (i.e., 0.1–2.0 Mb) corresponding to inbreeding events dating back to 2,800–144 years BP.

**Table 1:** Timing of inbreeding events based on ROH lengths assuming a generation time of 6 years^27^

ROH length (Mb)	Generations BP	Years BP
<0.1	older than 480	older than 2880
≥0.5	up to 96	up to 576
≥2	up to 2.4	up to 144
≥5	up to 9.6	up to 58
≥10	up to 4.8	up to 29
≥30	up to 1.6	up to 10
≥70	up to 0.7	up to 4

### Genetic load

**Figure 3:**
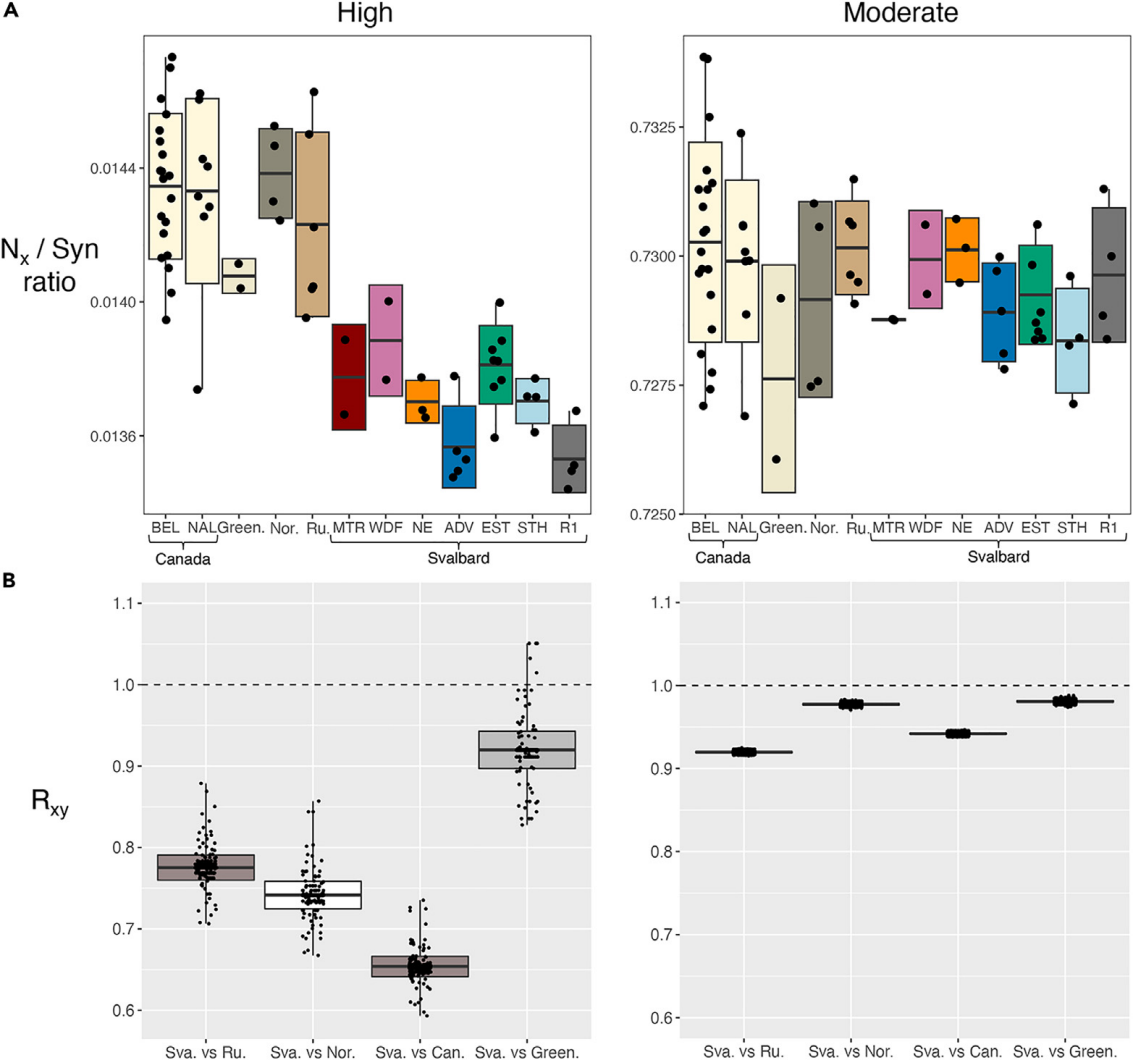
Geographical comparison of genetic load in coding regions for five *Rangifer tarandus* populations (A) Ratio of the number of high and moderate impact (N_x_) to synonymous variants per individual. Svalbard reindeer is divided into seven subpopulations. (B) R_xy_ of derived alleles for high- and moderate-impact variants for Svalbard reindeer relative to other populations. R_xy_ < 1 or >1 corresponds to a decrease or an increase in allele frequency, respectively, in population x relative to population y (Can. = Canada; Green. = Greenland; Nor. = Norway; Ru. = Russia, Sva. = Svalbard). Horizontal lines within boxplots depict the mean, bounds of boxes represent the standard deviation, and vertical bars represent minima and maxima.

Estimates of load based on the ratio of high impact to synonymous variants, as well as R_xy_, revealed a significantly lower load in Svalbard reindeer relative to larger mainland populations (p = 1 × 10^−6^; Figures 3A, 3B, and S3). After removing variants fixed in all reindeer populations, there was an average of 42 high-impact variants per individual in Svalbard compared to an average of 50 in the larger populations (Canada, n = 56; Western Greenland, n = 52; Norway, n = 54; Russia, n = 50; Table S1). However, there was no significant difference in load estimates for moderate-impact variants (p > 0.764; Figure 3A), only a slight decrease in their allele frequencies for Svalbard relative to Russia and Canada, and an even lower decrease relative to Norway and Western Greenland (Figure 3B). Furthermore, the Western Greenland individuals showed a reduction in high-impact load relative to larger mainland reindeer populations, but a higher load relative to Svalbard reindeer when considering the ratio of high impact to synonymous variants and R_xy_ (Figures 3A and 3B).

We found a total of 259 genes with high-impact variants and 8,340 genes with moderate-impact variants across the five reindeer populations (Table S2). When considering Svalbard reindeer alone, we found 41 genes with high-impact variants, including 23 genes with premature STOP codons (i.e., stop-gained) and 1,416 genes with moderate-impact variants (Table S2).

### Population genomic simulations

To assess the plausibility of our interpretation of the temporal dynamics of load and examine the impact of the past founder event, geographical isolation, and the recent bottlenecks on Svalbard reindeer genome-wide diversity, we performed genome-informed simulations for scenarios recapitulating the past population history of the subspecies. We tested the effects of the founder and long-term post-colonization population sizes under the hypothesis that a small number of individuals would induce the largest purging effect. Based on past demography reconstruction and historical and contemporary population size estimates,^29^ we simulated a large ancestral mainland population of 50,000 individuals from which founders (K_anc_founder_ = 25 to 500) colonized the archipelago 7,000 years BP. This new population then increased and stabilized to ∼1,000, 2,000, or 5,000 individuals 6,900 years BP and subsequently increased to ∼20,000 individuals 1,200 years BP until the severe bottleneck (N = 1,800^29^) 200 years BP. Finally, from 1925 onward, the population recovered to 20,000 individuals.^29^ To examine the dynamics of load in a large and stable population that only experienced the recent overharvesting bottleneck, we also simulated a null scenario with a long-term, constant population size of ∼20,000 individuals since the isolation of the archipelago.

**Figure 4:**
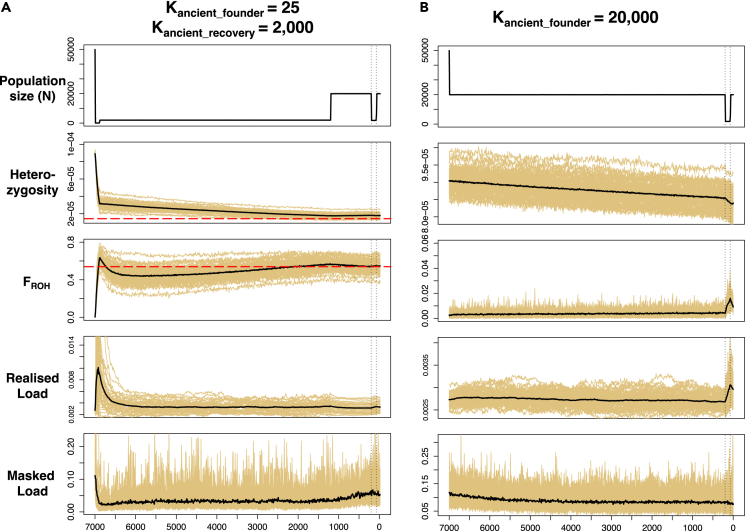
Forward simulations modeling the impact of the ancient founder event and the recent bottleneck on genetic variation for Svalbard reindeer (A) A scenario depicting a severe founder effect (K_anc_founder_ = 25) 7,000 years BP, a population increase and stabilization 6,900 years BP (K_anc_recovery_ = 2,000), an increase 1,200 years BP (K_hist_ = 20,000), a recent bottleneck 200 years BP (K_rec_bot_ = 1800^29^), and a final recovery 100 years BP (K_rec_recovery_ = 20,000^29^). The red dashed horizontal lines represent empirical estimates for mean heterozygosity and F_ROH_ in modern Svalbard reindeer. (B) A null scenario with a large founder population size (K_anc_founder_ = 20,000) only including the recent decline and a recovery 200 and 100 years BP, respectively. Panels show the temporal changes in population size (N), mean heterozygosity, mean inbreeding (F_ROH_), mean realized, and masked load. The recent bottleneck and the recovery at time 200 and 100 years BP are depicted with vertical dotted black lines. The black line represents the mean over 30 replicates (colored lines).

The scenarios simulating the most severe founder effects with K_anc_founder_ ∼25–100 followed by a post-colonization recovery to 1,000–2,000 individuals were the most consistent with the empirical data, showing a ∼77% reduction in heterozygosity and substantial increases in inbreeding (F_ROH_ = 0.6–0.8; Figures 4A and S4–S6) over the past 7,000 years. Furthermore, inbreeding steadily increased, and heterozygosity continued to decrease, after the demographic recovery starting 6,900 years BP. In contrast, scenarios assuming larger founder population sizes ≥ 100 and post-colonization recovery to 2,000–5,000 revealed only ∼33% reductions in heterozygosity and only slight increases in inbreeding (F_ROH_ = 0.1–0.2; Figures S4–S6). The null scenario with K_anc_founder_ = 20,0000 only showed an obvious increase in F_ROH_ and reduction in heterozygosity associated with the recent bottleneck (Figure 4B).

The overall dynamics of genetic load was similar across scenarios and depicted an initial increase in realized load (and thus in inbreeding depression) coinciding with the founder effect, but this increase was strongest for a K_anc_founder_ ≤ 50 (Figures 4A and S4–S6). This increase was followed by a decline over the following ∼500 years, and there was little change in realized load until the present day. Only the null scenario with K_anc_founder_ = 20,000 showed a marked increase in realized load associated with the recent bottleneck (Figures 4B and S3). The masked load declined within the first ∼200 years after the founder event in all scenarios, and this reduction was the sharpest for the scenarios with K_anc_founder_ ≤ 50 (i.e., ∼75%–80% fold vs. ∼50% for K_anc_founder_ = 250–500). There was also a slight increase in masked load over the past ∼1,000 years in all scenarios, except for K_anc_founder_ = 20,000 (Figure 4B).

**Figure 5:**
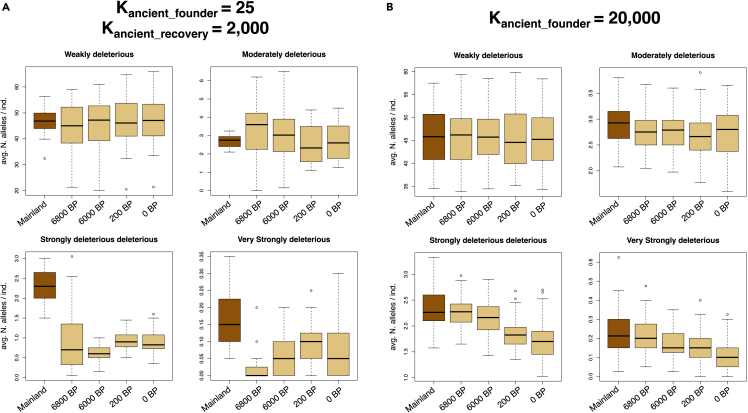
Forward simulations modeling the impact of the ancient founder event and the recent bottleneck on deleterious variants for Svalbard reindeer (A) A scenario depicting a severe founder effect. (B) A null scenario with a large founder population size as described in Figure 4. Figures show the average number of weakly, moderately, strongly, and very strongly deleterious alleles per individual for a large Eurasian mainland reindeer population and Svalbard reindeer at different time points after divergence (i.e., 6,800, 6,000, 200, and 0 years BP). Horizontal lines within boxplots depict the median, bounds of boxes represent the standard deviation and whiskers represent minima and maxima. Results of alternative scenarios are shown in Figures S4–S6.

When considering variants of different impacts, smaller founder population sizes always showed a greater reduction in moderately to very strongly deleterious variation relative to a larger population (Figures 5A and S4–S6). Most of this reduction occurred within 200 to 1,000 years from the founder event. However, scenarios with a K_anc_founder_ ≤ 100 showed the most rapid reduction. Nevertheless, the recent bottleneck induced a slight to moderate increase in deleterious variation for strongly to very strongly deleterious variation since 6,000 years BP (Figure 5B), except for K_anc_founder_ = 20,000 (Figure S3). Weakly deleterious variation did not vary between the two populations in any scenario (Figures S4–S6).

## Discussion

We used a comparative genomics approach and forward simulations to examine the dynamics of genetic load in the Svalbard reindeer, an endemic subspecies that is adapted to the High-Arctic island environment and that has experienced both ancestral and recent bottlenecks. We confirm that the Svalbard reindeer is the most inbred subspecies of *Rangifer tarandus*^30^ investigated so far. Our empirical results are consistent with simulations and indicate that high inbreeding associated with a strong ancestral founder effect and long-term geographical isolation facilitated the purging of its genetic load. Importantly, our results suggest that an initial period of inbreeding depression and low genetic diversity did not preclude the subspecies rapid adaptation to the extreme High-Arctic island environment of Svalbard.

Population structure was consistent with previous analyses using a subset of the data for Canadian caribou^31^ and Svalbard reindeer^27^^,^^32^ and further showed a close relationship between Russian and Svalbard reindeer.^25^^,^^28^ The divergence time between Novaya Zemlya and Svalbard estimated at ca. 6,200 years BP is consistent radiocarbon dating and molecular evidence^24^^,^^25^^,^^28^ that suggest reindeer colonized the archipelago between 5,000 and 11,000 years BP via Novaya Zemlya and the Franz Josef Land archipelagos. This stepping-stone dispersal scenario is further supported by ancient reindeer remains indicating that Franz Josef Land could have been colonized between ca. ≥ 6,400 and 1,300 years BP.^34^ Since low-lying parts of Svalbard were ice-free only 12,000 years BP,^35^ an earlier colonization can be excluded. However, the 6,000–8,000 years BP period coincides with intense glacier retreat and a warmer climate than present day.^36^ Furthermore, our past demographic reconstructions indicate that the Svalbard reindeer population remained stable after the colonization of the archipelago until 1,000–2,000 years BP, when the metapopulation recovered to several tens of thousands of individuals. It is unlikely that this increase in population size was driven by a higher plant species diversity and vegetation productivity, as sedimentary ancient DNA analyses show higher diversity in older sediments from warmer periods (i.e., >4300 years BP).^37^^,^^38^ Instead, this period coincides with Late Holocene glacial re-advances on Svalbard (4,000–0 years BP),^36^ which may have favored admixture among genetically distinct populations toward the center of Spitsbergen (e.g., WDF, ADV),^27^^,^^32^^,^^39^ a glacial refugium where more vegetation was likely to persist. This may also explain the biologically unrealistic high N_e_ values for WDF (∼20,000)^29^ using GONE.

The naturally recolonized subpopulation at Mitrahalvøya (MTR) and the Southern Spitsbergen (STH) subpopulation showed the highest inbreeding (F_ROH_), and the reintroduced subpopulation R1 showed similar values to its source subpopulation of Adventdalen (ADV). While our F_ROH_ estimates are overall higher and showed a larger proportion of long ROH (≥ 2 Mb) compared to those of Burnett et al.,^27^ they are not directly comparable since the latter were not based on hard genotype calls and high-coverage genomic data. Furthermore, F_ROH_ estimates and inference of timing of past demographic events based on ROH lengths should be taken with caution and may not accurately reflect the exact timing of inbreeding events due to uncertainties around generation times, unequal data coverage and types, and variant calling approaches (i.e., hard calls vs. genotype likelihoods).^40^ Nevertheless, and importantly, our results show that both long-term isolation and the recent bottleneck have contributed to the high inbreeding in Svalbard reindeer in comparison with other reindeer subspecies.

Our estimates of genetic load in the Svalbard reindeer population are consistent with theoretical predictions^11^^,^^13^ and a growing number of empirical studies showing that long-term isolation and the associated high inbreeding facilitates the reduction of genetic load through purging (e.g.,^14^^,^^15^^,^^16^^,^^17^^,^^18^^,^^19^). Our results also indicate that while highly deleterious variation is purged, purifying selection is less efficient at removing moderately deleterious variation, due to their lower selection coefficients.^11^^,^^13^ Thus, the threat of inbreeding depression, through the expression of such deleterious variation over the long term, remains. For instance, Alpine ibex nearly went extinct through overhunting in the early 1900s and has lost ∼80% of its pre-bottleneck mitochondrial genetic variation.^41^ Moreover, following this bottleneck to ∼100 individuals, several reintroductions and founder events may have facilitated the purging of highly deleterious variation while moderately and weakly deleterious mutations accumulated.^15^

Simulations have proven useful in recent years as they allow researchers to assess the plausibility of results interpretations as well as to predict future demographic and genomic trajectories of populations under contrasting demographic scenarios.^42^ Here, while the overall dynamics of load was similar among scenarios, smaller founder population sizes of 25–50 individuals followed by a stable population size of 1,000–2,000 consistent with our past N_e_ estimate of ∼500 based on the SMC++, were always associated with a greater increase in inbreeding and reduction in genetic load. Thus, they were the most consistent with our empirical data. It is worth noting that there is no certainty around the initial number of founders and that the founder population sizes in our simulation (i.e., 25 to 500) are meant to represent the sum of several smaller founder events over a period of ca. 100 years. Furthermore, we cannot exclude that other fluctuations occurred over the following millennia in association with glacial expansion or retreat.^37^ Nevertheless, our results support that the reduction in masked load may have occurred rapidly within 200 to 1,000 years of the founder event, meaning that the subspecies most likely experienced an initial period of inbreeding depression after colonization.^13^ Our simulations also clearly illustrate the dynamics of genetic load in this isolated island population, where a portion of the masked load is converted into realized load as inbreeding increases, which leads to an overall reduction in total load.^11^^,^^13^ In contrast, simulations showed only minimal change in masked load associated with the recent bottleneck ca. 200 years BP. The short demographic decline over ca. 100 years and a rapid rebound most likely prevented the occurrence of a more substantial reduction and further supports that a small number of founders combined with a small long-term population size were the main drivers of genetic load reduction in Svalbard reindeer.

Consistent with theory and other studies (e.g., ibex,^15^ Montezuma quail,^43^ mountain gorilla^44^), our simulations also reveal that weakly deleterious alleles are not reduced under any scenario due to their lower selection coefficients and lower impact on fitness.^11^^,^^13^ This further illustrates the possible contribution of weakly deleterious variation to inbreeding depression even after purging has occurred.^11^^,^^13^ However, there is a clear negative relationship between the size of the founder population and the extent of reduction in load (from moderately to highly deleterious alleles). This further supports a scenario of a small founder population size for Svalbard reindeer (i.e., ∼25–50 individuals), which is also the most likely, given the distances crossed^35^ and consistent with a stepping-stone dispersal.^25^ This small founder population size may have been further constrained by reduced suitable habitat at the time of colonization as well as subsequent sea-level rise that isolated the population on the newly formed archipelago.

Unlike the Svalbard reindeer, barren-ground caribou of Western Greenland (*Rangifer tarandus groenlandicus*), the second most inbred subspecies in our dataset, has retained a high genetic load. This suggests that purifying selection has been less efficient or that this decline was too recent for the effects of purging to be detectable. Western Greenland caribou have high inbreeding levels reflecting both historical and recent bottlenecks^45^^,^^46^ and consistent with a population decline from 100,000 to 8,000 between 1970 and 1980 through the combined effects of climate, overgrazing, and hunting.^46^ This more recent decline contrasts with the long-term isolation of Svalbard reindeer. Western Greenland genomes illustrate a different dynamics of load, in which a sudden population decline is more likely to empower drift, weaken purifying selection, and increase the risk of inbreeding depression.^2^^,^^12^ This effect has been shown in a number of species that have experienced severe recent declines (e.g., Swedish wolf,^9^^,^^47^ Arctic fox,^48^ Soay sheep^49^).

It is worth noting that there are some caveats regarding the different estimates of genetic load estimation and how load translates into impacts on fitness. First, since the selection coefficient *s* and dominance coefficient *h* are typically unknown, it is challenging to make assumptions on the actual fitness effects of mutations, unless candidate genes linked to fitness have previously been identified (e.g., dwarfism in California condor^50^). Secondly, annotation quality can bias results due to annotation of variants in pseudogenes or incomplete annotations.^13^ Furthermore, various metrics have been used as proxies for genetic load,^11^^,^^21^ and we focus here on the total numbers of variants since both variants in homozygous and heterozygous state will affect the fitness and viability of the whole population in future generations.^13^ Nevertheless, while the link between genetic load (i.e., measured as lethal equivalents or number of deleterious variants) and fitness effects needs to be examined with fitness data, genetic estimates can be used as a proxy for population health and provide essential knowledge on the ability of populations to purge deleterious variation.^11^^,^^13^

While it is essential to consider the dynamics of genetic load when assessing population viability, it is also crucial to consider how bottlenecks can affect adaptive variation (e.g., loss through drift, fixation of maladapted variant^51^). A recent study on temporal genomics of Svalbard reindeer indicates that historical overhunting led to shifts in frequency through genetic drift in regions containing genes potentially important for fitness, including circadian rhythm regulation, fat storage, and immune response.^39^ Consequently, both temporal changes in load and in the frequency of adaptive variation should be taken into account when assessing the genomic consequences of severe population bottlenecks.^3^^,^^52^

One concern associated with purging of load in small populations is that the associated reduction in genetic diversity could also reduce the population’s adaptive potential (e.g.,^53^). In spite of an initial period of increase in realized load and inbreeding depression, long-term high inbreeding, reduced genetic diversity, and strong purifying selection, this isolated reindeer subspecies successfully adapted to the environmental conditions of the High Arctic. Svalbard reindeer show a number of morphological and physiological adaptations typical of other island mammalian species (e.g., reduced body size,^54^ lack of predator avoidance^55^) as well as adaptations to arctic environments (e.g., maintenance of circadian rhythm throughout dark winters,^55^^,^^56^^,^^57^ low metabolism,^58^ fat deposition, and programmed winter anorexia^55^^,^^56^ and insulation^59^) and diet (e.g., ability to digest bryophytes^22^^,^^60^). Assuming a generation time of six years,^27^ this corresponds to ca. 1,200 generations. Accelerated morphological evolution in island mammals is well known in other mammals (e.g., dwarfism).^61^^,^^62^ However, the timing of such changes vary widely among taxa. For instance, Sicilian elephants (*Palaeoloxodon falconeri*) experienced up to 99% reduction in mass over a 200,000–400,000 years period after divergence from their mainland ancestor.^63^ In contrast, there are examples of more rapid evolution. Channel Island deer mice (*Peromyscus maniculatus*) subspecies have experienced significant changes in morphology including reduction in depth of braincase; total length, tail length, and hindfoot length became smaller over a period of 90 years, at a rate exceeding those estimated from paleontological records.^64^ Nevertheless, examples of local adaptation involving several presumably polygenic morphological, physiological, and behavioral traits on such a short timescale as shown for Svalbard reindeer are rare. This demonstrates that, at least in some scenarios, small populations can still adapt to strong environmental pressures in spite of severe loss of diversity and strong purifying selection.

With the current rapid rate of climate change in the Arctic,^65^ the availability of reindeer habitat in Svalbard is also rapidly changing.^66^^,^^67^^,^^68^ The possible expansion of suitable vegetation means that Svalbard reindeer populations could increase.^29^^,^^69^ However, owing to its adaptations specific to arctic environments, milder conditions may negatively impact the fitness of Svalbard reindeer, and it is unclear whether populations might decline above a certain temperature optimum.^70^ To understand whether the subspecies can adapt to rapidly changing conditions, it is thus crucial to understand the genomic basis of such adaptations and examine the temporal changes in putatively selected alleles in response to previous warming-cooling cycles of the Holocene. While our results show that reduced genetic diversity has not prevented the Svalbard reindeer from rapidly adapting to its novel environment, it is unlikely that it will be able to adapt to the current unprecedented rapid Arctic warming. This also applies to other terrestrial species that have a limited ability to migrate and track their habitat and that may thus face an increased risk of extinction.^71^^,^^72^ Moreover, the Arctic is expected to be sea-ice free as early as 2050.^73^ Since Svalbard reindeer dispersal is characterized by crossing sea ice, it is likely that peripheral populations, which already have the lowest abundance^29^ and genetic diversity^27^^,^^32^ will become further isolated. Consequently, the source-sink dynamics may be severely altered and may threaten the viability of the subspecies as a whole, especially given the low probability of further immigration under near-future Arctic sea-ice scenarios.

### Limitations of the study

Estimating the timing and tempo of genome-wide changes in natural populations is challenging. While our data provide strong support for purging of genetic load and for long-term reduced genetic diversity in Svalbard reindeer, we can only speculate on the tempo of and interplay between purifying selection and local adaptation through positive selection. Thus, the main limitation resides on our ability to test whether purifying and positive selection occurred simultaneously or sequentially. An ancient DNA approach would be the most appropriate to examine the two processes and track genomic changes in real time.

## STAR★Methods

### Key resources table

**Table:** 

REAGENT or RESOURCE	SOURCE	IDENTIFIER
**Deposited data**		

*Rangifer Tarandus Platyrhynchus* assembly and annotations	GenBank (https://www.ncbi.nlm.nih.gov); ENA (https://www.ebi.ac.uk/)	GCA_949782905.1; PRJEB60852
*Odocoileus hemionus* assembly and annotations	https://www.ncbi.nlm.nih.gov	GCA_002197005.1
Resequencing data	Burnett et al. 2022^27^	PRJEB57293
	Weldenegodguad et al. 2020^30^	PRJEB317216
	Taylor et al. 2020^31^	PRJNA634908
	This study	PRJEB61721

**Software and algorithms**		

hifiasm v0.16.1	Cheng et al.^74^	https://github.com/lh3/hifiasm-meta
BWA v0.7.17	Li and Durbin^75^	http://bio-bwa.sourceforge.net/
YaHS v1.1a	Zhou et al.^76^	https://github.com/c-zhou/yahs/
Merqury v1.3	Rhie et al.^77^	https://github.com/marbl/merqury/
BUSCO v5.3.1	Manni et al.^78^	https://busco.ezlab.org/busco_userguide.html
GRIT rapid curation suite	Howe et al.^79^	https://gitlab.com/wtsi-grit/rapid-curation
PretextView v0.2.5	https://github.com/wtsi-hpag/PretextView	https://github.com/wtsi-hpag/PretextView
Repeatmodeller v1.0.11	http://www.repeatmasker.org	http://www.repeatmasker.org
RepeatMasker v4.0.7	http://www.repeatmasker.org	http://www.repeatmasker.org
Generode pipeline	Kutschera et al.^80^	https://github.com/NBISweden/GenErode
fastp v0.22.0	Chen et al.^81^	https://github.com/OpenGene/fastp
SAMtools v1.12	Li et al.^82^	https://sourceforge.net/projects/samtools/files/samtools/1.3/
Picard MarkDuplicates v2.26.6	http://broadinstitute.github.io/picard/	http://broadinstitute.github.io/picard/
GATK v3.4.0	McKenna et al.^83^	https://gatk.broadinstitute.org/hc/en-us
bcftools v1.8	Li^84^	http://www.htslib.org/
BEDtools v2.29.2	Quinlan^85^	https://bedtools.readthedocs.io/en/latest/
Minimap2	Li^86^	https://github.com/lh3/minimap2
PLINK v1.9	Chang et al.^87^	https://www.cog-genomics.org/plink2/
SMC++ v1.15.2	Terhorst et al.^88^	https://github.com/popgenmethods/smcpp
GONE	Santiago et al.^89^	https://github.com/esrud/GONE
mlRho v2.7	Haubold et al.^90^	http://guanine.evolbio.mpg.de/mlRho/
R	R Core Team^91^	https://www.r-project.org/
SNPeff v4.3	Cingolani et al.^92^	http://snpeff.sourceforge.net/index.html
Cufflinks v 2.2.1	Trapnell et al.^93^	http://cole-trapnell-lab.github.io/cufflinks/
SLiM 4.0	Haller and Messer^94^^,^^95^	https://messerlab.org/slim/

### Resource availability

#### Lead contact

Further information and requests for resources and reagents should be directed to and will be fulfilled by the lead contact, Nicolas Dussex (nicolas.dussex@gmail.com).

#### Materials availability

This study did not generate new unique reagents.

### Experimental model and subject details

#### Materials and methods

##### Data collection

Our dataset comprised previously sequenced and newly-sequenced genomes for five wild populations of *Rangifer tarandus*, making for a total of 133 genomes. We obtained genome data for Canadian (n = 28) and Western Greenland (n = 2) caribou (PRJNA634908^31^) as well as from Russian reindeer (n = 2; PRJEB317216^30^). We also combined newly-sequenced and published genome data from Burnett et al.^27^ for Svalbard reindeer (n = 91; PRJEB57293; PRJEB61721) and resequenced genomes from Russian (n = 6) and Norwegian reindeer (n = 4; Table S1).

DNA was extracted from ∼20 mg skin or muscle tissue using a DNeasy Blood & Tissue Kit (Qiagen, Hilden, Germany) and randomly sheared into short fragments of 400 bp. Genomic libraries were prepared either in-house using a blunt-end, single-tube protocol for double-stranded DNA (BEST v1.1)^96^ as described in Burnett et al.^27^ or at Novogene UK using our DNA extracts. Genomic libraries were sequenced on the Illumina NovaSeq 6000 platform using 2 × 150 bp chemistry at Novogene, UK or on an Illumina HiSeq 4000 using 2 × 150 bp chemistry at the NTNU Genomics Core Facility (See supplemental information).

### Methods details

#### *De-novo* genome sequencing and assembly

High molecular weight DNA was extracted from skeletal muscle tissue that was obtained from a male Svalbard reindeer collected from Colesdalen Valley, Nordenskiöld Land, Svalbard. HiFi and Hi-C libraries were prepared using a PacBio library preparation kit 2.0 (PacBio) and Dovetail Omni-C kit. Libraries were sequenced on a quarter Illumina NovaSeq S4 flowcell with 2 × 150 bp paired end mode at the Norwegian Sequencing Center (See supplemental information).

HiFi reads were assembled using hifiasm v0.16.1^74^ and Hi-C reads were aligned to each scaffolded assemblies (i.e., Haplotype 1 and 2) using BWA-MEM v0.7.17.^75^ The resulting BAM file was used to scaffold the two assemblies using YaHS v1.1a^76^ with default options. Merqury v1.3^77^ was used to assess the completeness and quality of the genome assemblies and BUSCO v5.3.1^78^ was used to assess the completeness of the genome assemblies with the “mammalia_odb10” dataset. The assemblies were manually curated using the GRIT rapid curation suite^79^ and the PretextView v0.2.5 (https://github.com/wtsi-hpag/PretextView, last accessed April 5, 2023).

Repeats were identified using Repeatmodeller v1.0.11 and repeatmasker v4.0.7 [Smit, A.F.A. and Hubley, R. (2008–2015) RepeatModeler Open-1.0, http://www.repeatmasker.org] as part of the GenErode bioinformatics pipeline.^80^

#### Genome data mapping

Raw data trimming, mapping to the *de-novo* Svalbard reindeer assembly (Haplotype 1) and variant calling was done using the GenErode bioinformatics pipeline.^80^ Briefly, adapter trimming was done with fastp v0.22.0.^81^ Reads were mapped using BWA-MEM v0.7.17.^75^ Read sorting was done using SAMtools v1.12,^82^ duplicates removal with picard MarkDuplicates v2.26.6 (http://broadinstitute.github.io/picard/), and read realignment around indels using GATK IndelRealigner v3.4.0.^83^

We called variant using the mpileup command of bcftools v1.8,^84^ filtering out variants using a minimum depth of coverage (DP4) of ∼⅓ (i.e., 5X) of the average depth of coverage, and base quality QV ≥ 30. Indels and SNPS within 5 bp of indels were removed. We also filtered out SNPs in heterozygous state that were not in an allelic balance (i.e., number of reads displaying the reference allele/depth) of <0.2 and >0.8 in order to avoid biases caused by contamination, mapping or sequencing error. After merging all individual vcf files, we excluded scaffolds linked to X and Y chromosomes and masked repeats with BEDtools v2.27.1.^85^

We selected 68 high-coverage genomes (i.e., ≥ 10X) from the five populations for heterozygosity, inbreeding and genetic load estimates (Table S1) and obtained 26,734,956 SNPs. After filtering for missing data with bcftools we retained 2,501,253 SNPs. We also selected 91 Svalbard genomes (i.e., ≥ 4X) for the demographic analyses and obtained a total of 1,495,188 high-quality SNPs when retaining SNPs called in all individuals.

For the genetic load analysis, which requires calling of variants relative to an ancestral allele or an outgroup,^21^ we re-mapped the same 68 genomes to *Odocoileus hemionus* (Odocoileus_hemionus_HiC, https://www.dnazoo.org/assemblies/Odocoileus_hemionus) using the same approach as described above. We used Minimap2^86^ to perform a synteny analysis against red deer (*Cervus elaphus;* GenBank: GCA_002197005.1) and identify the X chromosomes (i.e., HiC_scaffold_35). After filtering for missing data and sex chromosomes, we obtained a multi-individual vcf file comprising 4,768,837 SNPs.

### Quantification and statistical analyses

#### Population structure and past demography

To identify the main genetic clusters in our dataset (n = 133) and check for consistency with previous studies,^27^^,^^31^^,^^32^ we performed a Principal Component Analysis (PCA) in PLINK v1.9.^87^ Throughout the manuscript, we refer to ‘populations’ based on the geographical origin of samples as well as on the clustering results shown here and in these previous studies. Genomes of Canadian caribou were divided into Euro-Beringian (BEL) and North American clusters (NAL)^31^ and Svalbard genomes into eight subpopulations (i.e., six wild and two reintroductions^27^; Table S1).

We reconstructed the past demography of Svalbard reindeer (n = 91) using two different approaches. First, we estimated past changes in N_e_ over the recent past (i.e., ∼100,000 years BP), by using SMC++ v1.15.2.^88^ This approach relies on Sequential Markov Coalescent (SMC) simulations from unphased genome data from multiple genomes. We used a substitution rate of 1.06 × 10e^−8^ per site per generation^33^ and generation time of six years,^27^ and inferred the past demography for each Svalbard reindeer subpopulation using the ‘estimation validation’ approach with --em-iterations 5000, and --thinning 1300, --regularization-penalty 6, --polarization-error of 0.5, --ftol of 1e-7, --c 1000000 and --xtol of 1e-7.

We also estimated population divergence between Svalbard and Russia (Novaya Zemlya) to infer the timing of colonisation of the archipelago using the same parameters as above. We first reconstructed the past demography for each population separately. Secondly, we estimated the joint SFS between two populations using the smc++ vcf2smc command. Finally, we calculated the marginal estimate of joint demography to estimate pairwise population divergence using the smc++ split command. For this analysis, we performed 50 bootstrap replicates for each population and for 6 chromosomes to reduce computational time and estimate the mean and 5^th^ and 95^th^ percentiles.

Secondly, we reconstructed the past demography of reindeer over the past 200 generations using GONE^89^ which estimates changes in N_e_ calculated as the geometric mean over 40 independent estimates from the observed spectrum of linkage disequilibrium (LD). We only retained the 34 largest autosomal chromosomes and used the following parameters: PHASE = 2; cMMb = 1.04^97^; DIST = 1; NGEN = 2000; NBIN = 400; MAF = 0.0; ZERO = 1; maxNCHROM = 85; maxNSP = 50000; REPS = 40; threads = −99. We reduced the hc value from the default of 0.05 to 0.01to avoid biases caused by recent immigration as suggested by Santiago et al.^89^ We performed 30 runs for each of the eight genetic clusters identified by Burnett et al.^27^

#### Heterozygosity and inbreeding

We estimated heterozygosity and inbreeding for our high coverage genomes dataset (n = 68). We first used mlRho v2.7^90^ to estimate the individual mutation rate (θ), which approximates the genome-wide heterozygosity measured as the number of heterozygous sites per 1,000 bp. We down-sampled each genome to the average coverage of the genome with lowest coverage (i.e., 10X), filtered out bases with quality (-Q) <30, mapped sequencing reads with mapping quality (-q) <30 and positions with root-mean-squared mapping quality (MQ) < 30 from the historical and modern bam files. We also filtered out sites with depth <⅓ (i.e., 5X) of the average depth of coverage and higher than 2X the average coverage across all our genomes to avoid false heterozygous sites due to structural variation and erroneous mapping.

We identified runs of homozygosity (ROH) using the sliding-window approach implemented in PLINK v1.9. We estimated inbreeding coefficients (F_ROH_) by dividing the sum of all ROH by the size of the genome (autosomes only). We used the following parameters: a sliding window size of 100 or 1000 SNPs (*homozyg-window-snp 100/1000*); no more than 1 or 3 heterozygous sites per window to assume a window as homozygous (*homozyg-window-het 1/3*); at least 5% of all windows including a given SNP to define the SNP as being in a homozygous segment (*homozyg-window-threshold 0.05*); a homozygous segment was defined as a ROH if the segment included ≥25 SNPs (*homozyg-snp 25*) and covered ≥100 kb (*homozyg-kb* 100); the minimum SNP density was one SNP per 50 kb (*homozyg-density 50*); and the maximum distance between two neighboring SNPs was ≤1,000 kb (*homozyg-gap 1,000*). Finally, we set the value at 750 heterozygous sites within ROH (*homozyg-het 750*) in order to prevent sequencing errors to cut ROH. We statistically compared heterozygosity and F_ROH_ among populations using Tukey HSD tests in R.^91^

Using the length of ROH, we also inferred the timing of inbreeding by solving *g* = 100/(2*rL*),^98^ where *g* corresponds to the number of generations back in time, *L* to the length of ROH in Mb, and *r* to the recombination rate. We used an *r* of 1.04 cM/Mb estimated in red deer^99^ and a generation time of 6 years.^27^ Table 1 shows the inferred times based on ROH lengths.

To estimate mutational load, we used SnpEff v4.3^92^ to annotate synonymous and non-synonymous nucleotide substitutions in coding regions using the data mapped to and gene annotation for *O. hemionus* (n = 68) in order to reduce reference and annotation bias (e.g.,^14^^,^^100^). After removing gene models with in-frame STOP codons, missing START and terminal STOP codons (-J option) and genes labeled as pseudogenes (--no-pseudo option) with Cufflinks v2.2.1,^93^ we obtained a total of 22,736 genes. We identified three categories of variants: a) Synonymous; b) Moderate: non-disruptive variants that might change protein effectiveness; c) High: variants assumed to have high (disruptive) impact on protein, probably causing protein truncation, loss of function or triggering nonsense mediated decay and including stop gained codons, splice donor variant and splice acceptor, start codon lost.^92^ We also excluded intergenic (-no-intergenic) and intron (-no-intron) variants. For each variant category, we recorded the number of homozygous and heterozygous variants and summed the total number of variants. We then corrected for potential mapping biases arising from different sample types (i.e., batch effects associated with different datasets and unequal distance to outgroup) by calculating the ratio of deleterious variants (High and Moderate impact) to Synonymous SNPs, following the approach of Xue et al.^44^ We compared the differences in load among populations using Tukey HSD in R.

To take into account the frequency of variants in each population, we also calculated the R_xy_ ratio for High and Moderate impact variants and for each population pair following Xue et al.^44^ We used a random number intergenic SNPs corresponding to the number of each impact type for standardisation, which makes R_xy_ robust against sampling effects and population substructure.^44^ An R_xy_ equal to 1 corresponds to no change in frequency between two populations, whereas R_xy_ < 1 or >1 corresponds to a decrease or an increase in frequency in population x relative to population y, respectively. We used a jack-knife procedure in R to estimate the variance in the R_xy_ ratio.

#### Population genomic simulations

We performed forward genome-informed simulations in SLiM 4.0.^94^^,^^95^ We used a non-Wright-Fisher model (nonWF) which allows for overlapping generations and where each cycle corresponds to a year. Also, the probability of an individual surviving from one year to the next is given by its absolute fitness, which ranges from 0 to 1 and which is determined by its genetic composition. Population size N is an emergent parameter controlled by carrying capacity (K) and is the outcome of a stochastic process of reproduction and viability selection. If N > K, the absolute fitness is rescaled downward by the ratio of K/N. Therefore, these models did not allow for population growth beyond K but instead allow for N to fluctuate around K.

To avoid the fitness of all individuals increasing to 1 in case of severe decline and to allow for viability selection and impacts of inbreeding depression, density-dependent selection was rescaled following Robinson et al.^101^ by drawing the new individual fitness as min(K/N, 1.0).

We created scenarios recapitulating the population history of the Svalbard reindeer based on the known species history^29^ and the demographic history inferred here based on genomic data with SMC++ and GONE. We considered five distinct demographic events with instantaneous changes in population size. After 500,000 years of burnin for a large mainland source population (K_source_ = 50,000), we simulated: (1) an ancient bottleneck corresponding to the founder event of the Svalbard population 7,000 years BP based on the oldest radiocarbon dated antlers (M. Le Moullec, B. B. Hansen & M. Martin, *unpubl. data*) for several founder population sizes (K_anc_founder_: 25, 50, 100, 250, 500), (2) a population recovery 6,900 years BP (K_anc_recovery_ = 1,000, 2,000 and 5,000), (3) a population increase 1,200 years BP (K_hist_ = 20′000), (4) a recent decline resulting from overhunting 200 years BP (K_rec_bot_: 1,800^29^) and, (5) a recent recovery 100 years BP (K_modern_ = 20,000^29^; Table S3). As a comparison, we also ran a null scenario with larger founder population sizes (K_anc_founder_: 20,000) that only included the recent decline and a recovery at 200 and 100 years BP, respectively.

Reproduction was modeled with the simplifying assumptions of a first age at reproduction of 1 (i.e., females giving birth when turning 2) and a conservative maximum of 13, and a harem-like reproduction system with only 50% of males allowed to reproduce (Hansen *pers. comm.*) and a reproductive output ranging from 1 to 8 offspring, with the probabilities declining with offspring number (Table S3), reflecting the higher reproductive success of a few males.^102^ The model assumed a maximum longevity of 16 years (Hansen *pers. comm.*) and age-specific mortality assuming the following probabilities^103^: Calves = 0.2; 1–14 years = 0.05; 15 years = 0.74; 16 years = 1.0 (Table S3).

We simulated 6 chromosomes containing each 500 genes of 1750 bp long following Robinson et al.^101^ We randomly generated deleterious (non-synonymous) mutations in exonic regions at a ratio of 2.31:1 to neutral (synonymous) mutations.^104^ For selection coefficients (*s*) of non-synonymous mutations we used distributions based on estimates in humans^105^ to model very strongly, strongly, moderately and weakly deleterious mutations as well as lethal mutations and using a gamma distribution a mean *s* = −0.01314833 and shape = 0.186 (Table S3). For dominance coefficients (*h*), we assumed an inverse relationship between *h* and *s*^106^^,^^107^ with *h* = 0.0 for very strongly deleterious mutations (*s* < −0.1), *h* = 0.01 for strongly deleterious mutations (−0.1 ≤ *s* < −0.01), *h* = 0.1 for moderately deleterious mutations (−0.01 ≤ *s* < −0.001), and *h* = 0.4 for weakly deleterious mutations (*s* > −0.001). For neutral mutations *s* was set to 0. We used a mutation rate of 1.76e^−9^ mutations/site/year based on Chen et al.^33^ (i.e., 1.06e^−8^ mutations/site/generation) and assuming a generation time of six years.^27^ For recombination rate, we assumed no recombination within genes, a rate of 1e^−3^ between genes, and free recombination between chromosomes.

Summary statistics included population size (N), mean heterozygosity, mean F_ROH_ (>0.1 Mb), the mean number of each non-synonymous mutation category (i.e., weakly to very highly deleterious deleterious) as well as mean realised load (i.e., reduction in fitness due to segregating and fixed deleterious mutations^11^) and mean masked load (i.e., the quantity of recessive deleterious variation concealed in heterozygotes^11^) as estimated in Kyriazis et al.^6^ based on a sample of 30 individuals every 10 years. For all scenarios we ran a total of 30 replicates each starting with a different seed.

## Data Availability

• Genome Assembly and annotations: GCA_949782905.1, BioProject: PRJEB60852; Resequencing data (ENA BioProject): PRJNA634908, PRJEB317216, PRJEB61721. • Code for data processing and analysis, and simulation are deposited to Github: https://github.com/ndussex/Reindeer_genome_erosion.git.

## Supplemental information

Document S1. Methods S1 and Figures S1–S6

Table S1. Sampling information and estimates of inbreeding, heterozygosity and load in 133 reindeer, related to Figures 1–3

Table S2. Allele frequencies for High and Moderate impact variants. Fixed variants in all populations were removed, related to Figure 3

Table S3. Demographic and genomic parameters used in the SLiM simulations, related to Figures 4–5 and STAR Methods
