# Supplementary material for: Adaptation to the High-Arctic island environment despite long-term reduced genetic variation in Svalbard reindeer
Source: iScience. 2023 Sep 3;26(10):107811. doi: 10.1016/j.isci.2023.107811 (PMC10514459; doi:10.1016/j.isci.2023.107811)
Supplement: Document S1. Methods S1 and Figures S1–S6 [file mmc1.pdf]

iScience, Volume *26*

Supplemental information

Adaptation to the High-Arctic island environment despite long-term reduced genetic variation in Svalbard reindeer

Nicolas Dussex, Ole K. Tørresen, Tom van der Valk, Mathilde Le Moullec, Vebjørn Veiberg, Ave Tooming-Klunderud, Morten Skage, Benedicte Garmann-Aarhus, Jonathan Wood, Jacob A. Rasmussen, Åshild Ø. Pedersen, Sarah L.F. Martin, Knut H. Røed, Kjetill S. Jakobsen, Love Dalén, Brage B. Hansen, and Michael D. Martin

Supplemental information

Adaptation to the High-Arctic island environment despite long-term reduced genetic variation in Svalbard reindeer

Nicolas Dussex, Ole K. Tørresen, Tom van der Valk, Mathilde Le Moullec, Vebjørn Veiberg, Ave Tooming-Klunderud, Morten Skage, Benedicte Garmann-Aarhus, Jonathan M.D. Wood, Jacob A. Rasmussen, Åshild Ø. Pedersen, Sarah L.F. Martin, Knut H. Røed, Kjetill S. Jakobsen, Love Dalén, Brage B. Hansen, Michael D. Martin

Methods S1: *De novo* genome assembly and annotation and resequencing data details, related to STAR Methods.

Sampling and isolation of genomic DNA

A male reindeer was harvested for scientific purposes in October 2021 in Colesdalen Valley, Nordenskiöld Land, Svalbard (78°04’29’’ N, 15°22’51’’ E) under permit 16/01632-40 granted by the Governor of Svalbard. A sample of skeletal muscle tissue of approximately 5 mm^3 was collected from the chest region using a sterile scalpel at an ambient temperature of -10 °C. The sample was stored at -80 °C (or on dry ice during transport) until DNA extraction. DNA isolation for PacBio long read sequencing was performed using Circulomics Nanobind CBB BIG DNA kit and protocol according to manufacturer’s recommendations (Circulomics, now PacBio company). Quality check of amount, purity and integrity of isolated DNA was performed using Qubit BR DNA quantification assay kit (Thermo Fisher), Nanodrop (Thermo Fisher), and Fragment Analyser (DNA HS 50kb large fragment kit, Agilent Tech.), respectively.

Library preparation and sequencing for *de-novo* assembly.

Before PacBio HiFi library preparation, DNA was purified an additional time using PacBio Ampure Beads (1:1 ratio). Then 7,5 µg of purified HMW DNA was sheared to approx. 15-20 kbp large fragments using the Megaruptor3 (Diagenode) in 200 µl volume. For library preparation, 5 µg of fragmented DNA was used following PacBio protocol for HiFi library prep using SMRTbell® express template prep kit 2.0. Final HiFi library was size-selected with a 10 kbp cut-off using a BluePippin instrument (Sage Biosciences) before sequencing on, in total, three SMRT cells on the PacBio Sequel II instrument at the Norwegian Sequencing Centre. We performed all QC steps with the same instrumentation as explained above.

Hi-C library was prepared using the Arima High Coverage Hic (HiC+) kit, following the manufacturer’s recommendations (document part number A160162v01) and starting from 50 mg fresh frozen muscle tissue. Final library quality was assayed using instrumentation described above as well as qPCR using the Kapa Library quantification kit for Illumina (Roche Inc.), before sequencing with other libraries on a quarter Illumina NovaSeq S4 flowcell with 2*150 bp paired end mode at the Norwegian Sequencing Centre.

De-novo genome assembly and annotation

KMC v3.1.2rc1[1] was used to count *k*-mers of size 21 in the PacBio HiFi reads. GenomeScope v2.0[2] was run on the *k*-mer histogram output from KMC to get estimates of genome size, heterozygosity and repetitiveness. HiFiAdapterFilt v2.0.0[3] was applied on the HiFi reads to remove possible remnant PacBio adapter sequences. The filtered HiFi reads were assembled using hifiasm v0.16.1[4] with Hi-C integration resulting in a pair of haplotype-resolved

assemblies, hap1 and hap2. Unique *k*-mers in each assembly was identified using meryl v1.3.0 (https://github.com/marbl/meryl), and used to create two sets of Hi-C reads, one without any k-mers occurring uniquely in hap1 and the other without k-mers occurring uniquely in hap2. *K*-mer filtered Hi-C reads were aligned to each scaffolded assemblies using BWA-MEM v0.7.17[5] with -5SPM options. The alignments were sorted based on name using Samtools 1.15.1[6], then Samtools fixmate was applied, before default sorting and applying samtools markdup. The resulting BAM file was used to scaffold the two assemblies using YaHS v1.1a[7] with default options. The UniVec Database was used to screen against putative retained vector sequences, such as adapters in the scaffolded assemblies, and any adaptor sequences found were masked using bedtools v2.30.0 maskfasta. FCS-GX v0.2.2 (https://github.com/ncbi/fcs) was used to search for contamination. If a contaminant was found on the start or end of a sequence, the sequence was trimmed using a combination of samtools faidx, bedtools complement and bedtools getfasta. If the contaminant was internal, it was masked using bedtools maskfasta. MitoHiFi v2.2[8] was used to identify the mitochondrial genome in contigs and reads. Merqury v1.3[9] was used to assess the completeness and quality of the genome assemblies by comparing to the *k*-mer content of the Hi-C reads. BUSCO v5.3.1[10] was used to assess the completeness of the genome assemblies by comparing against the expected gene content in the *mammalia* lineage. Gfastats v1.2.2[11] was used to output different assembly statistics of the assemblies. The assemblies were manually curated using the GRIT rapid curation suite[12] and the PretextView v0.2.5 (https://github.com/wtsi-hpag/PretextView, last accessed April 5, 2023).

For annotation, RNA-seq data for *Rangifer tarandus* was downloaded from the BioProject PRJEB44094 and mapped against the assemblies using HiSat2 v2.2.1[13] and StringTie2 v2.2.1[14] was used to assemble these into transcripts. AGAT v1.0 (https://zenodo.org/record/7255559) scripts agat_sp_keep_longest_isoform.pl and agat_sp_extract_sequences.pl were used on the GRCh38 assembly and annotation to generate one protein (the longest isoform) per gene. Miniprot v0.5[15] was used to align the proteins to the curated assemblies. UniProtKB/Swiss-Prot release 2022_03[16] in addition to the vertebrata part of OrthoDB v10[17] were also aligned separately to the assemblies. RED v2018.09.10[18] was run via redmask (https://github.com/nextgenusfs/redmask) on the assemblies to mask repetitive areas. GALBA (https://github.com/Gaius-Augustus/GALBA: f4aaeca; [15,19–22]) was run with the human proteins using the miniprot mode on the masked assemblies. The funannotate-runEVM.py script from Funannotate v1.8.13 (https://zenodo.org/record/4054262) was used to run EvidenceModeler v1.1.1[23] on the alignments of human proteins, UniProtKB/Swiss-Prot proteins, vertebrata proteins and the predicted genes from GALBA in addition to the transcripts from StringTie2. The resulting predicted proteins were compared to the protein repeats that Funannotate distributes using DIAMOND v2.0.15 blastp[24] and the predicted genes were filtered based on this comparison using AGAT. The filtered proteins were compared to the UniProtKB/Swiss-Prot release 2022_03 using DIAMOND blastp to find gene names and InterProScan v5.47-82[25] was used to discover functional domains. AGATs agat_sp_manage_functional_annotation.pl was used to attach the gene names and functional annotations to the predicted genes. EMBLmyGFF3 v2.2[26] was used to combine the fasta files and GFF3 files into a EMBL format for submission to ENA.

A total of 32-fold coverage in Pacific Biosciences single-molecule HiFi long reads and 56-fold coverage in Arima Hi-C reads were generated and assembled with hifiasm in Hi-C integration mode, resulting in two haplotype-separated assemblies. The contigs generated were scaffolded with the chromosome conformation Hi-C data with YaHs, resulting in scaffold N50 of 66 Mbp (haplotype 1) and 65 Mbp (haplotype 2). Manual assembly curation corrected 9 and 7 misjoins and made 108 and 79 joins in haplotypes 1 and 2, respectively. 34 autosomes were identified in haplotype 1 (numbered by length). X and Y chromosomes were identified based

on comparisons with an elk genome assembly (*Cervus canadensis;* accession GCF_019320075.1) added to haplotype 1. The final assemblies have total lengths of 2,970 Mbp and 2,830 Mbp, respectively, with 96.3 % and 94.1 % complete BUSCO genes using the mammalian lineage set and contig N50 of 22.5 Mbp (haplotype 1) and 25.5 Mbp (haplotype 2). Haplotype 1 has a k-mer completeness of 96.5 %, haplotype 2 92.6 %, and 97.9 % together, compared to a k-mer database of the Hi-C reads. Further, haplotype 1 has an assembly consensus quality value (QV) of 48.8 and haplotype 2 of 49.7, where a QV of 40 corresponds to one error every 10,000 bp, or 99.99 % accuracy and 50 is one error every 100,000 bp or 99.999 % accuracy. 32,643 and 26,502 protein-coding genes were annotated in haplotype 1 and 2 respectively.

Sampling and resequencing

For each library, PCR reactions were prepared in 100 µL volumes containing 7 µL library template, a unique combination of indexed forward and reverse primers (each at 0.25 µM final conc.), dNTPs (each at 0.25 mM final conc.), 1 µL Herculase II Fusion DNA Polymerase (Agilent), Herculase II reaction buffer (1x final conc.), and molecular biology water. Based on their performance in a prior qPCR assay, libraries were given X=13−21 cycles of PCR during indexing. PCR consisted of holding libraries at 95 °C for 3 minutes, followed by X cycles of 95 °C for 20 seconds, 60 °C for 20 seconds, and 72 °C for 40 seconds. After the final cycle, libraries were held at 72 °C for five minutes and then cooled to 4 °C. Amplified libraries were purified and size-selected by combining 100 µL of SPRI beads[30] with 100 µL PCR product on a magnetic rack, washing twice with 200 µL 80% ethanol, and then eluting the DNA in 33 µL EB buffer after incubation at 37 °C for 10 minutes. The prepared libraries were quantified on an Agilent TapeStation 4200.

For genomic libraries prepared at Novogene UK, the genomic DNA was randomly sheared into short fragments with a target length of 350 bp. The obtained fragments were end-repaired, A-tailed, and ligated with Illumina adapters. The resulting libraries were PCR amplified, size selected, and purified using AMPure beads. Each library was quantified using real-time PCR and assessed using a Qubit fluorometer and Agilent BioAnalyzer 2100. Genomic libraries were sequenced at Novogene UK on the Illumina NovaSeq 6000 platform using 2x150bp chemistry or on the Illumina HiSeq 4000 platform using 2x150bp chemistry at the NTNU Genomics Core Facility.

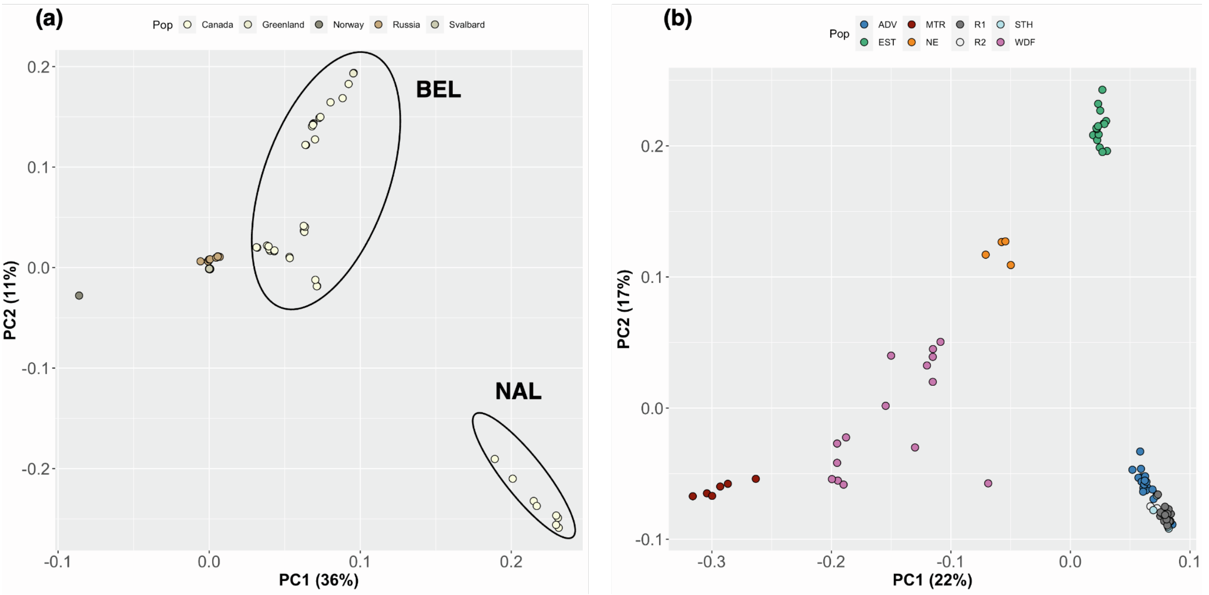

Figure S1. Principal component analysis for *Rangifer tarandus*, Related to Figure 1. (a) caribou and reindeer. Circles depict the Euro-Beringian (BEL) and North American lineages (NAL) as described in Taylor *et al.*[27]. **(b)** Svalbard reindeer only.

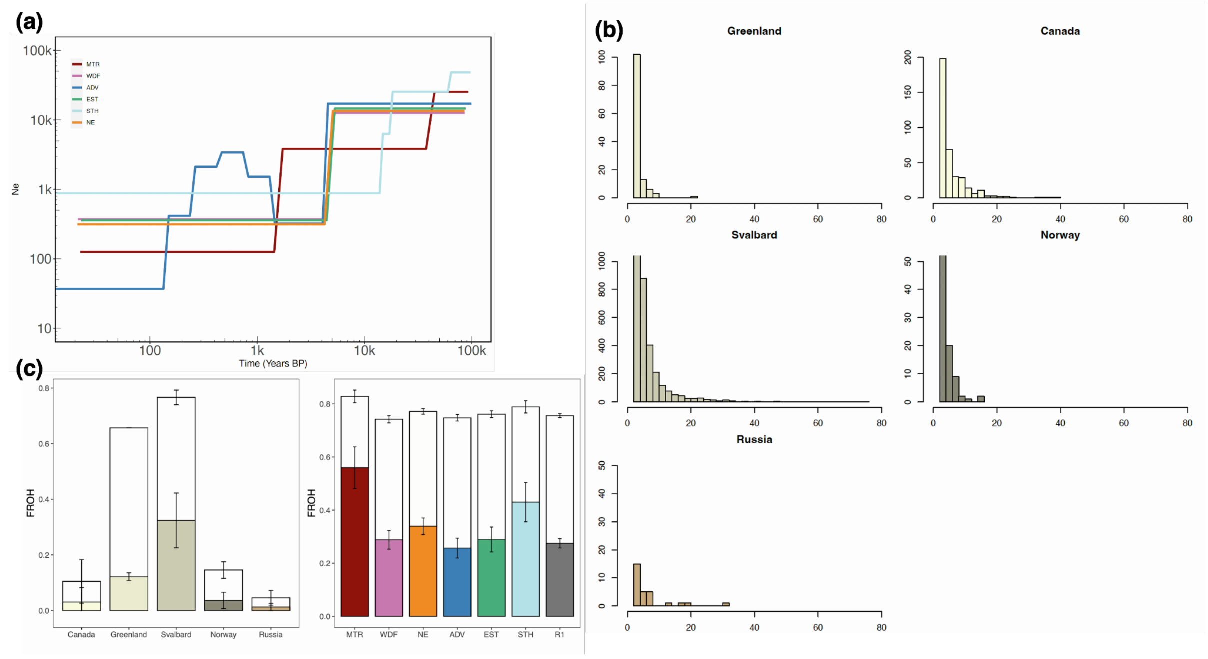

Figure S2. Past demography and inbreeding for Svalbard reindeer, Related to Figures 1 and 2. (a) Past demography of six Svalbard Reindeer subpopulations using SMC++ based on the mutation rate of *R. tarandus* (1.1 x 10e^{-8})[31] and generation time of six years[28]. **(b)** ROH (≥ 2Mb) distribution for five reindeer populations. Results are shown using the same parameters as for Fig. 2 (*homozyg-window-snp* 1000, *homozyg-window-het* 1). **(c)** Inbreeding coefficients (F_{ROH}) for five reindeer populations and seven Svalbard reindeer subpopulations based on Burnett *et al.*[28] clustering. Bars extending from the mean values represent the standard deviation. Complete bars show the proportion of genomes in ROH ≥100kb (i.e., background relatedness) and lower portions of bars show proportions in ROH ≥2Mb (i.e., recent inbreeding events). Results are shown as a comparison with Fig. 2 for more strict parameters: *homozyg-window-snp* 100, *homozyg-window-het* 3.

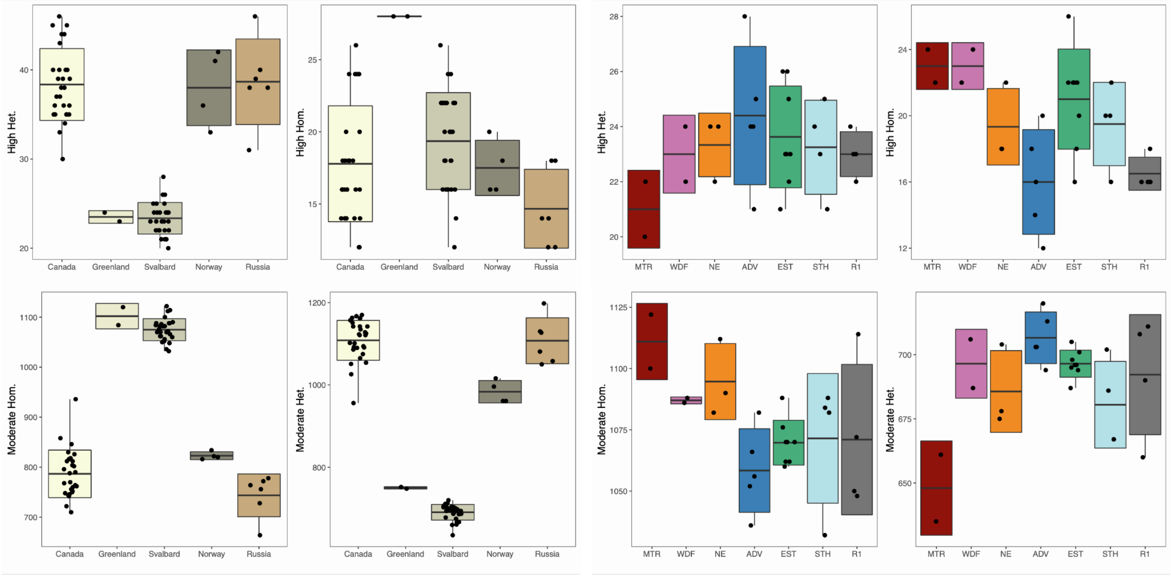

Figure S3. Genetic load for Svalbard reindeer, Related to Figure 3. Segregating High and Moderate impact variants stratified by heterozygous and homozygous state for five reindeer Holarctic populations and seven Svalbard reindeer subpopulations based on Burnett *et al.*[28] clustering.

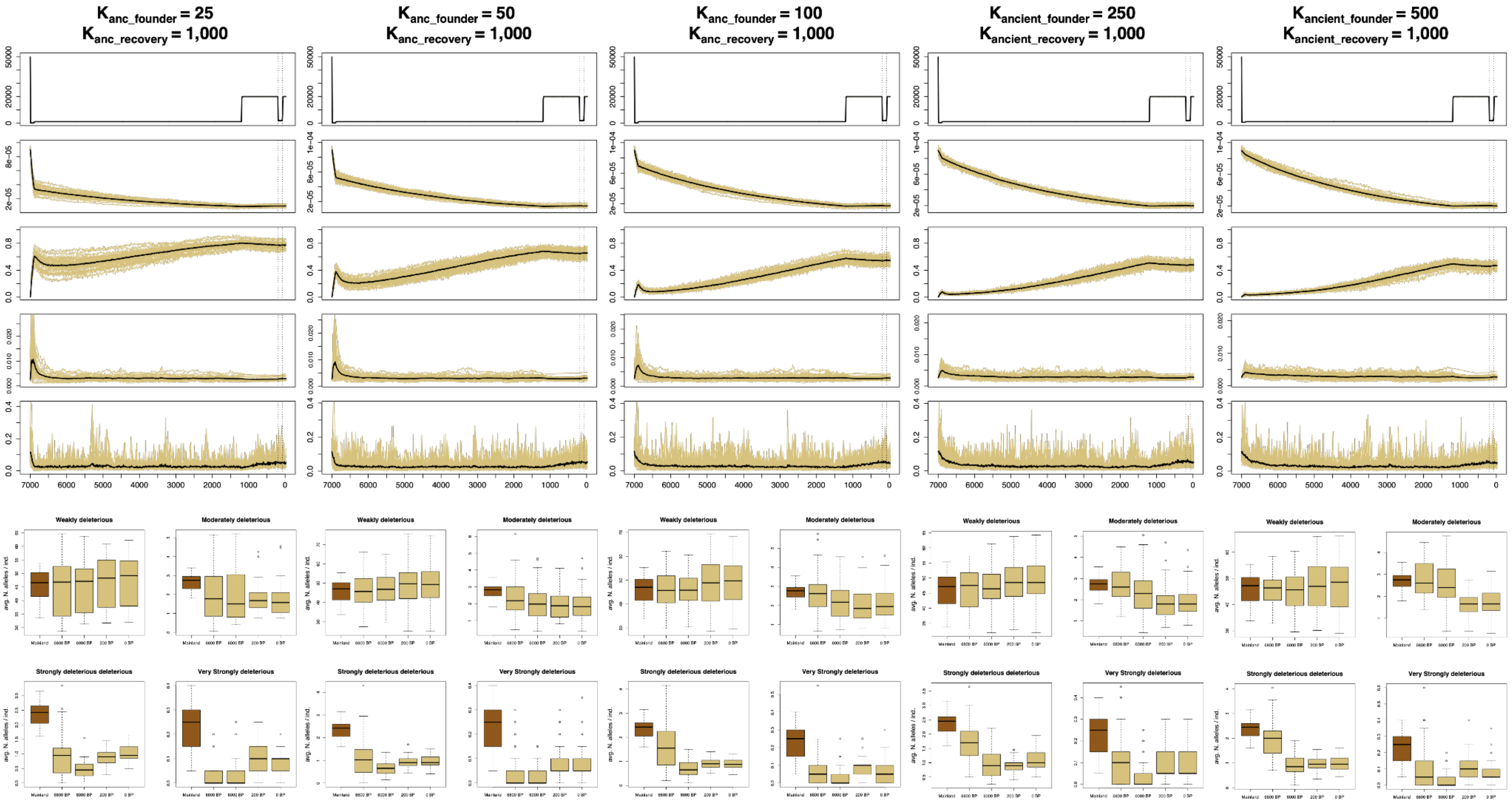

Figure S4. Changes in demography (N), heterozygosity, inbreeding (F_{ROH}), Realised and Masked load and the number of weakly, moderately, strongly and very strongly deleterious mutations over 7,000 years, Related to Figures 4 and 5. Simulations based on $K_{ancient\_founder}$ = 25 to 500 and $K_{anc\_recovery}$= 1,000.

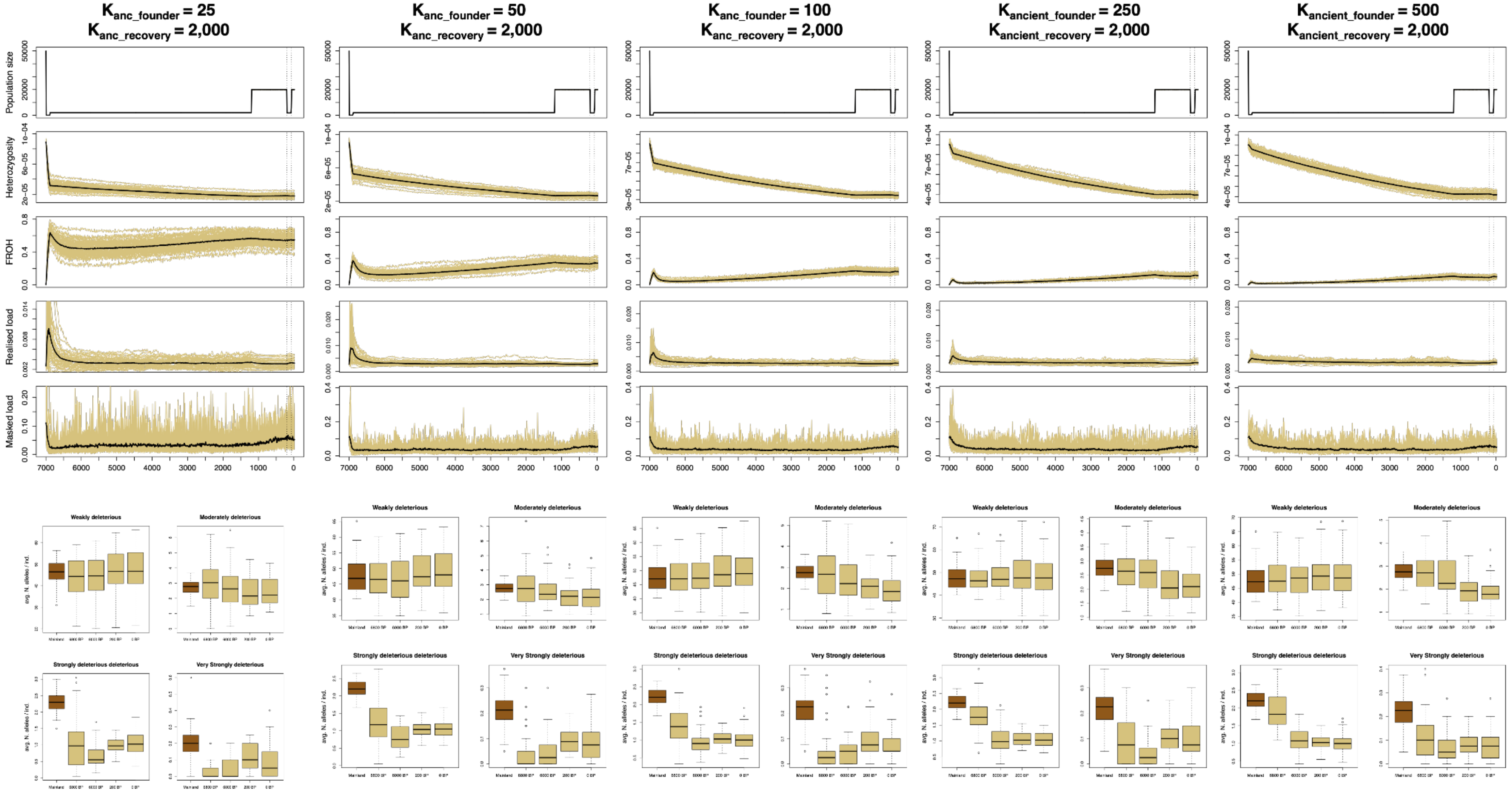

Figure S5. Changes in demography (N), heterozygosity, inbreeding (F_{ROH}), Realised and Masked load and the number of weakly, moderately, strongly and very strongly deleterious mutations over 7,000 years, Related to Figures 4 and 5. Simulations based on $K_{ancient\_founder}$ = 25 to 500 and $K_{anc\_recovery}$= 2,000.

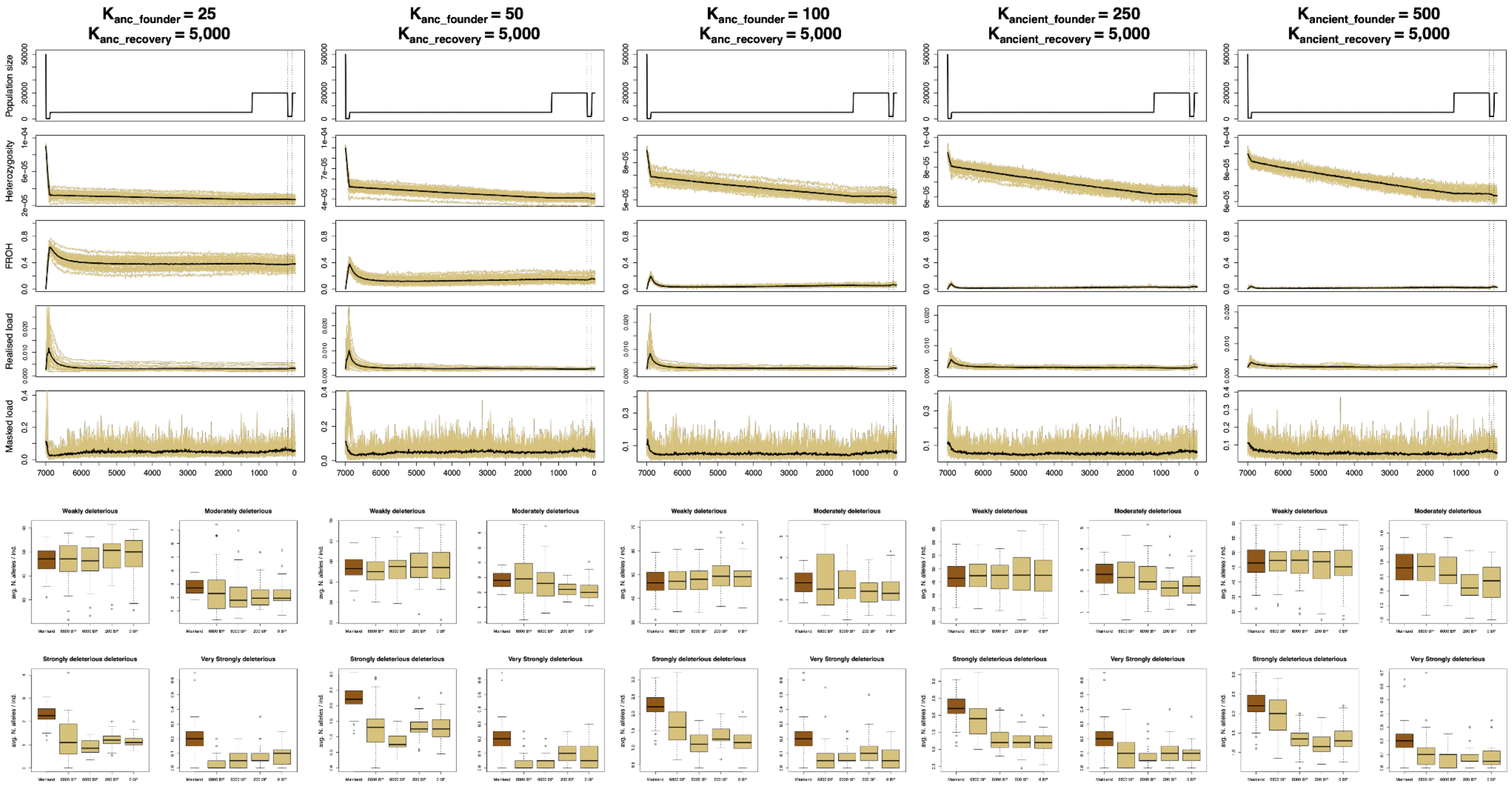

Figure S6. Changes in demography (N), heterozygosity, inbreeding (F_{ROH}), Realised and Masked load and the number of weakly, moderately, strongly and very strongly deleterious mutations over 7,000 years, Related to Figures 4 and 5. Simulations based on $K_{ancient\_founder}$ = 25 to 500 and $K_{anc\_recovery}$= 5,000.

References

1. Kokot, M., Dlugosz, M., and Deorowicz, S. (2017). KMC 3: counting and manipulating k-mer statistics. Bioinformatics *33*, 2759–2761.

2. Ranallo-Benavidez, T.R., Jaron, K.S., and Schatz, M.C. (2020). GenomeScope 2.0 and Smudgeplot for reference-free profiling of polyploid genomes. Nat. Commun. *11*, 1432.

3. Sim, S.B., Corpuz, R.L., Simmonds, T.J., and Geib, S.M. (2022). HiFiAdapterFilt, a memory efficient read processing pipeline, prevents occurrence of adapter sequence in PacBio HiFi reads and their negative impacts on genome assembly. BMC Genomics *23*, 157.

4. Cheng, H., Concepcion, G.T., Feng, X., Zhang, H., and Li, H. (2021). Haplotype-resolved de novo assembly using phased assembly graphs with hifiasm. Nat. Methods *18*, 170–175.

5. Li, H., and Durbin, R. (2010). Fast and accurate long-read alignment with Burrows-Wheeler transform. Bioinformatics *26*, 589–595.

6. Li, H., Handsaker, B., Wysoker, A., Fennell, T., Ruan, J., Homer, N., Marth, G., Abecasis, G., Durbin, R., and 1000 Genome Project Data Processing Subgroup (2009). The Sequence Alignment/Map format and SAMtools. Bioinformatics *25*, 2078–2079.

7. Zhou, C., McCarthy, S.A., and Durbin, R. (2023). YaHS: yet another Hi-C scaffolding tool. Bioinformatics *39*. 10.1093/bioinformatics/btac808.

8. Uliano-Silva, M., Ferreira, J.G.R., Krasheninnikova, K., Darwin Tree of Life Consortium, Formenti, G., Abueg, L., Torrance, J., Myers, E.W., Durbin, R., Blaxter, M., et al. (2023). MitoHiFi: a python pipeline for mitochondrial genome assembly from PacBio High Fidelity reads. bioRxiv, 2022.12.23.521667. 10.1101/2022.12.23.521667.

9. Rhie, A., Walenz, B.P., Koren, S., and Phillippy, A.M. (2020). Merqury: reference-free quality, completeness, and phasing assessment for genome assemblies. Genome Biol. *21*, 245.

10. Manni, M., Berkeley, M.R., Seppey, M., Simão, F.A., and Zdobnov, E.M. (2021). BUSCO Update: Novel and Streamlined Workflows along with Broader and Deeper Phylogenetic Coverage for Scoring of Eukaryotic, Prokaryotic, and Viral Genomes. Mol. Biol. Evol. *38*, 4647–4654.

11. Formenti, G., Abueg, L., Brajuka, A., Brajuka, N., Gallardo-Alba, C., Giani, A., Fedrigo, O., and Jarvis, E.D. (2022). Gfastats: conversion, evaluation and manipulation of genome sequences using assembly graphs. Bioinformatics *38*, 4214–4216.

12. Howe, K., Chow, W., Collins, J., Pelan, S., Pointon, D.-L., Sims, Y., Torrance, J., Tracey, A., and Wood, J. (2021). Significantly improving the quality of genome assemblies through curation. Gigascience *10*. 10.1093/gigascience/giaa153.

13. Kim, D., Paggi, J.M., Park, C., Bennett, C., and Salzberg, S.L. (2019). Graph-based genome alignment and genotyping with HISAT2 and HISAT-genotype. Nat. Biotechnol. *37*, 907–915.

14. Kovaka, S., Zimin, A.V., Pertea, G.M., Razaghi, R., Salzberg, S.L., and Pertea, M. (2019). Transcriptome assembly from long-read RNA-seq alignments with StringTie2. Genome Biol. *20*, 278.

15. Li, H. (2023). Protein-to-genome alignment with miniprot. Bioinformatics *39*. 10.1093/bioinformatics/btad014.

16. UniProt Consortium (2023). UniProt: the Universal Protein Knowledgebase in 2023. Nucleic Acids Res. *51*, D523–D531.

17. Kriventseva, E.V., Kuznetsov, D., Tegenfeldt, F., Manni, M., Dias, R., Simão, F.A., and Zdobnov, E.M. (2019). OrthoDB v10: sampling the diversity of animal, plant, fungal, protist, bacterial and viral genomes for evolutionary and functional annotations of orthologs. Nucleic Acids Res. *47*, D807–D811.

18. Girgis, H.Z. (2015). Red: an intelligent, rapid, accurate tool for detecting repeats de-novo on the genomic scale. BMC Bioinformatics *16*, 227.

19. Hoff, K.J., Lomsadze, A., Borodovsky, M., and Stanke, M. (2019). Whole-Genome Annotation with BRAKER. Methods Mol. Biol. *1962*, 65–95.

20. Hoff, K.J., and Stanke, M. (2019). Predicting Genes in Single Genomes with AUGUSTUS. Curr. Protoc. Bioinformatics *65*, e57.

21. Stanke, M., Schöffmann, O., Morgenstern, B., and Waack, S. (2006). Gene prediction in eukaryotes with a generalized hidden Markov model that uses hints from external sources. BMC Bioinformatics *7*, 62.

22. Buchfink, B., Xie, C., and Huson, D.H. (2015). Fast and sensitive protein alignment using DIAMOND. Nat. Methods *12*, 59–60.

23. Haas, B.J., Salzberg, S.L., Zhu, W., Pertea, M., Allen, J.E., Orvis, J., White, O., Buell, C.R., and Wortman, J.R. (2008). Automated eukaryotic gene structure annotation using EVidenceModeler and the Program to Assemble Spliced Alignments. Genome Biol. *9*, R7.

24. Buchfink, B., Reuter, K., and Drost, H.-G. (2021). Sensitive protein alignments at tree-of-life scale using DIAMOND. Nat. Methods *18*, 366–368.

25. Jones, P., Binns, D., Chang, H.-Y., Fraser, M., Li, W., McAnulla, C., McWilliam, H., Maslen, J., Mitchell, A., Nuka, G., et al. (2014). InterProScan 5: genome-scale protein function classification. Bioinformatics *30*, 1236–1240.

26. Norling, M., Jareborg, N., and Dainat, J. (2018). EMBLmyGFF3: a converter facilitating genome annotation submission to European Nucleotide Archive. BMC Res. Notes *11*, 584.

27. Rohland, N., and Reich, D. (2012). Cost-effective, high-throughput DNA sequencing libraries for multiplexed target capture. Genome Research *22*, 939–946. 10.1101/gr.128124.111.

28. Taylor, R.S., Manseau, M., Horn, R.L., Keobouasone, S., Golding, G.B., and Wilson, P.J. (2020). The role of introgression and ecotypic parallelism in delineating intraspecific conservation units. Mol. Ecol. *29*, 2793–2809.

29. Chen, L., Qiu, Q., Jiang, Y., Wang, K., Lin, Z., Li, Z., Bibi, F., Yang, Y., Wang, J., Nie, W., et al. (2019). Large-scale ruminant genome sequencing provides insights into their evolution and distinct traits. Science *364*. 10.1126/science.aav6202.

30. Burnett, H.A., Bieker, V.C., Le Moullec, M., Peeters, B., Rosvold, J., Pedersen, Å.Ø., Dalén, L., Loe, L.E., Jensen, H., Hansen, B.B., et al. (2022). Contrasting genomic consequences of anthropogenic reintroduction and natural recolonisation in high-arctic wild reindeer. bioRxiv, 2022.11.25.517957. 10.1101/2022.11.25.517957.
